# Exploring the Conformational Effects of *N*- and *C*-Methylation of *N*-Acylhydrazones

**DOI:** 10.1021/acsomega.5c01289

**Published:** 2025-04-21

**Authors:** Lucas Silva Franco, Marina Amaral Alves, Carlos Mauricio R. Sant’Anna, Isadora Tairinne de Sena Bastos, Regina Cely Rodrigues Barroso, Fanny Nascimento Costa, Fabio Furlan Ferreira, Carlos A. M. Fraga, Eliezer J. Barreiro, Lídia Moreira Lima, Daniel A. Rodrigues, Pedro de Sena M. Pinheiro

**Affiliations:** a Laboratório de Avaliação e Síntese de Substâncias Bioativas (LASSBio), Instituto de Ciências Biomédicas, Universidade Federal do Rio de Janeiro, Rio de Janeiro, RJ 21941-902, Brazil; b Instituto Nacional de Ciência e Tecnologia de Fármacos e Medicamentos (INCT-INOFAR), CCS, Universidade Federal do Rio de Janeiro, Cidade Universitária, Rio de Janeiro, RJ 21941-902, Brazil; c Walter Mors Institute of Research on Natural Products, Federal University of Rio de Janeiro (UFRJ), Rio de Janeiro, RJ 21941-599, Brazil; d Departamento de Química Fundamental, Instituto de Química, Universidade Federal Rural do Rio de Janeiro, Seropédica, RJ 23970-000, Brazil; e LabFisMed, State University of Rio de Janeiro, Physics Institute, Rio de Janeiro, RJ 20550-900, Brazil; f Center for Natural and Human Sciences, Federal University of ABC, Santo André, SP 09210-580, Brazil; g School of Pharmacy and Biomolecular Sciences (PBS), Royal College of Surgeons in Ireland, first Floor Ardilaun House Block B 111 St Stephen’s Green, Dublin D02 YN77, Ireland; h Department of Physics, State University of Feira de Santana, Feira de Santana, BA 44036-900, Brazil

## Abstract

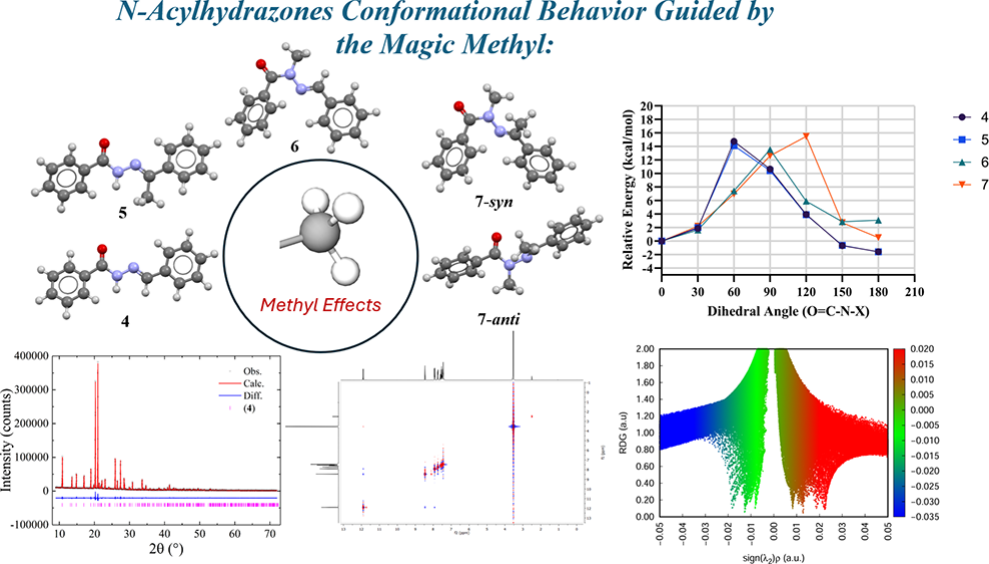

*N*-Acylhydrazones (NAH) are privileged structures in chemistry and medicinal chemistry. In this study, we describe the conformational effects of *N*- and *C*-methylated *N*-acylhydrazone derivatives, combining theoretical and experimental data analysis. Four *N*-acylhydrazone (NAH) derivatives (**4**–**7**) were synthesized and structurally characterized to investigate the impact of methylation on their conformational preferences and electronic properties. The structural characterization by NMR spectroscopy, including 2D techniques (HSQC, HMBC, and NOESY), confirmed the exclusive formation of (*E*)-diastereomers. Theoretical conformational analysis using density functional theory (DFT) calculations (CAM-B3LYP/6-31+G(d,p) with the C-PCM solvation model) revealed that *N*-methylation (**6**) significantly alters the preferred dihedral angle (O=C–N–X), inducing a shift from an antiperiplanar to a synperiplanar conformation. Notably, compound **7** showed two possible conformers in solution, *anti* and *syn* at the amide bond, and exhibited a greater deviation from planarity due to steric effects imposed by the two methyl groups, which disrupt conjugation within the NAH moiety. This was further supported by natural bond orbital (NBO) analysis, which demonstrated changes in electron density distribution, particularly at the carbonyl and imine carbons, correlating well with the calculated and experimental ^13^C NMR chemical shifts. Noncovalent interaction (NCI) analysis and powder X-ray diffraction provided additional evidence for these conformational trends, reinforcing the influence of methylation on NAH planarity. The findings highlight the steric and electronic consequences of methylation on NAH derivatives, which may have implications for their biological activity and molecular recognition properties.

## Introduction

The *N*-acylhydrazone (NAH) moiety is a versatile scaffold with applications in various branches of chemistry, notably in the design of bioactive substances^1^ and as electrophiles for the synthesis of nitrogen-containing compounds.^2^ NAHs are efficiently synthesized through the condensation of aldehydes or ketones with *N*-acylhydrazines, yielding crystalline compounds that can be purified by simple recrystallization.

The biological activity of different NAH derivatives has been investigated and reported in medicinal chemistry literature.^3−13^ The NAH subunit serves as a framework for agents with potent anticancer,^14,15^ antimicrobial,^5,6,10^ anti-inflammatory,^8,16^ antiplatelet,^17^ vasodilator,^18^ cardiac stimulant,^19,20^ and antiparasitic^21^ activities. The role of NAHs as ligands in medicinal inorganic chemistry has augmented the therapeutic potential and/or enhanced biological activity of various metal complexes.^22,23^

**Figure 1:**
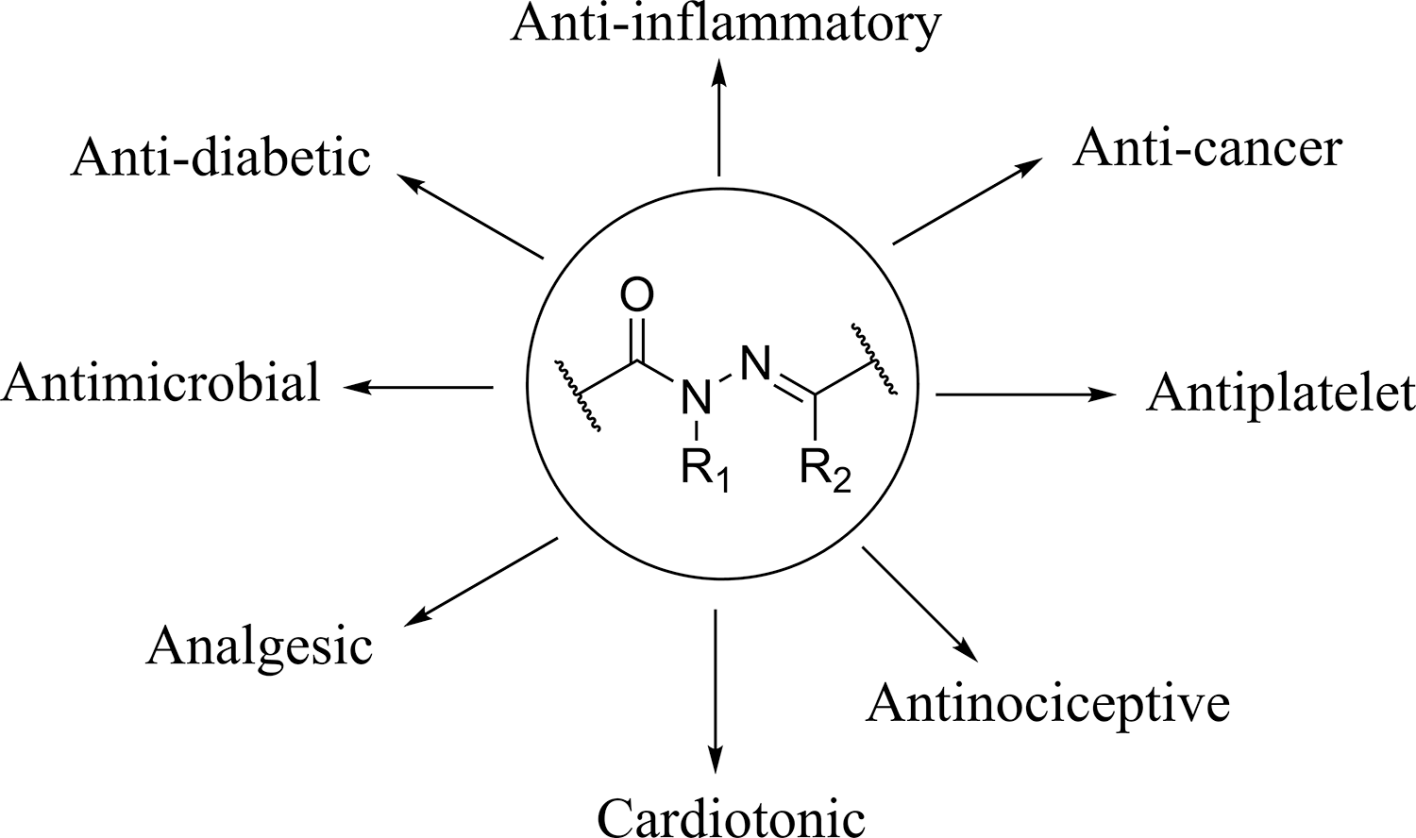
Bioactivities associated with the privileged NAH structure.

The NAH group, found in various compounds exhibiting diverse biological activities, is recognized as a privileged structure (Figure 1) with increasing applications in medicinal chemistry.^1,24^ Substitution at the R_1_ position (Figure 1) with alkyl groups is the most widely employed strategy when modifying the NAH functional group. *N*-methylation imparts unique properties by inducing conformational changes,^25^ which may result in a distinct pharmacological profile for the new series of compounds,^4,18,26^ as well as enhanced solubility and chemical stability.^27^

The modification of the R_2_ position (Figure 1) is far less explored in NAH chemistry, necessitating further discussion of how such modifications could alter the structure and properties of NAHs. Therefore, this study seeks to evaluate the conformational effects resulting from *N*- and *C-*methylation of the NAH framework, utilizing both experimental and theoretical data for a comprehensive analysis.

## Results and Discussion

**Scheme 1:**
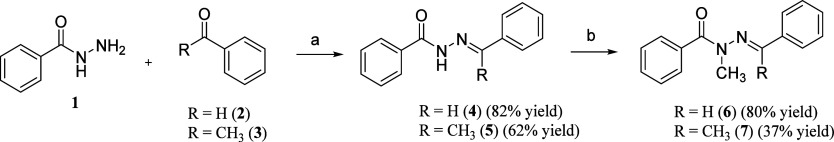
Synthesis of the NAH Derivatives **4**–**7** Studied in This Work

Four NAH derivatives were synthesized, as shown in Scheme 1. Benzohydrazide (**1**) was reacted with benzaldehyde (**2**) and acetophenone (**3**) via a condensation reaction under microwave conditions, furnishing (*E*)-*N′*-benzylidenebenzohydrazide (**4**) and (*E*)-*N′*-(1-phenylethylidene)benzohydrazide (**5**) with yields of 82 and 62%, respectively. Subsequently, *N*-alkylation of compounds **4** and **5** was carried out using methyl iodide in a basic medium (potassium carbonate) in acetone, leading to the formation of (*E*)-*N′*-benzylidene-*N*-methylbenzohydrazide (**6**) and (*E*)-*N*-methyl-*N′*-(1-phenylethylidene)benzohydrazide (**7**) with yields of 80 and 37%, respectively.

The characterization of the synthesized NAH derivatives was conducted by using ^1^H and ^13^C nuclear magnetic resonance (NMR) spectroscopy. The heteronuclear single quantum coherence (HSQC) and heteronuclear multiple bond correlation (HMBC) NMR experiments were employed to accurately correlate carbon and hydrogen signals, facilitating the structural elucidation of the synthesized compounds. The analysis of the ^1^H NMR spectra revealed that each compound was obtained as a single diastereomer. This conclusion was drawn from the presence of a single imine hydrogen signal (compounds **4** and **6**) or a single signal corresponding to the methyl group attached to the imine unit (compounds **5** and **7**), suggesting an (*E*) configuration. Previous studies^28^ have described this configuration for different bioactive *N*-acylhydrazone and *N*-methyl-*N*-acylhydrazone derivatives, as determined by ^1^H NMR and X-ray analyses.^4,26,29,30^ In addition, 2D NOESY experiments on all four NAH derivatives further confirmed the relative configuration (*E*) at the imine double bond of the NAH moiety (see Figures S5, S10, S15, and S20, Supporting Information).

To assess the significance of the conformational effects induced by the methylation of the NAH, we conducted a potential energy surface scan of the dihedral angle associated with the amide function (dihedral angle for O=C–N–X, where X = H or CH_3_). This analysis was performed using a hybrid exchange-correlation functional with the Coulomb-attenuating method CAM-B3LYP^31^ and a 6-31+G(d,p) basis set, employing the C-PCM solvent model^32,33^ for polar organic solvents (ε = 37.22) available in SPARTAN′24. The CAM-B3LYP/6-31+G(d,p) method is well suited for studying the conformational and electronic properties of *N*-acylhydrazone derivatives due to its proven accuracy in describing π-conjugated systems and electronic transitions. CAM-B3LYP incorporates long-range corrections, making it ideal for systems with extended conjugation, while the 6-31+G(d,p) basis set enhances the orbital description with polarization functions, capturing subtle electronic effects. This method has been successfully applied to NAH derivatives, providing reliable structural and spectroscopic predictions.^34−36^

**Figure 2:**
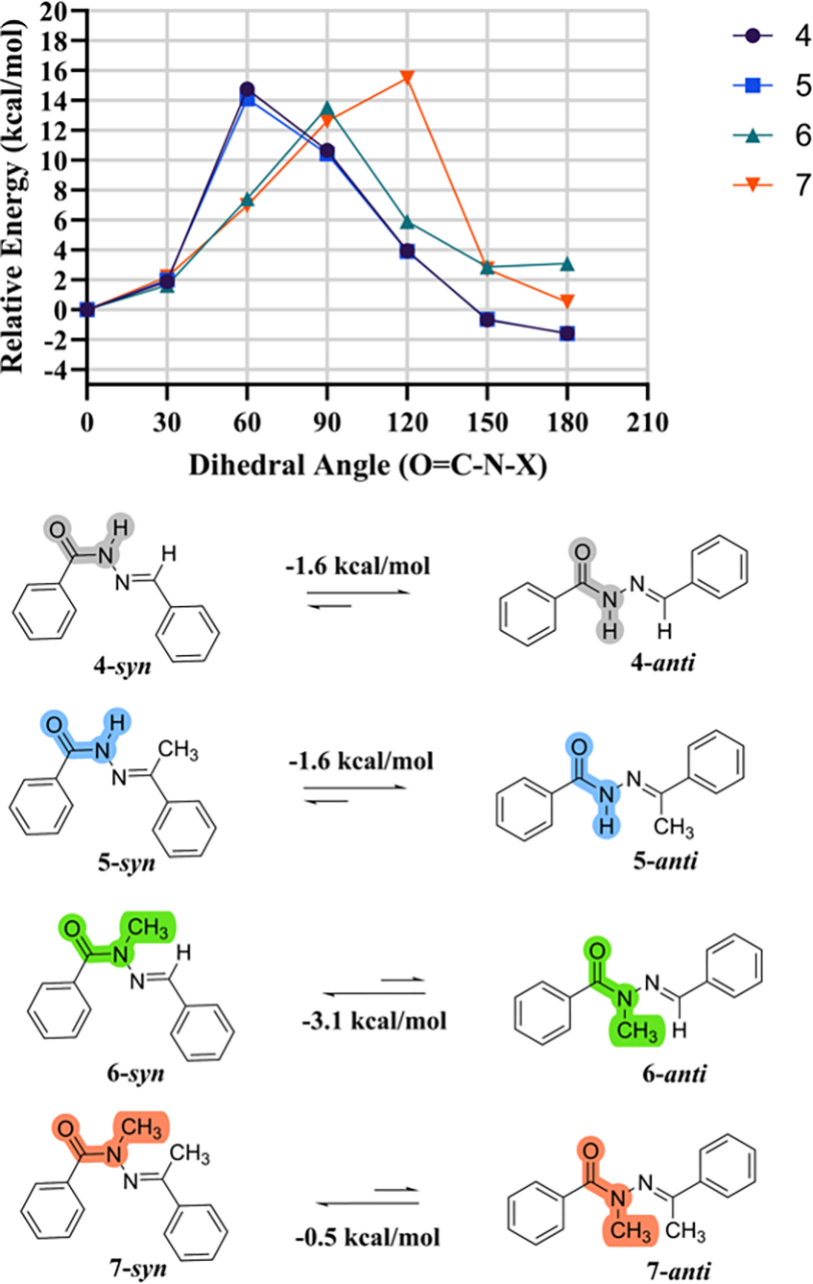
Potential energy curves of the compounds evaluated through CAM-B3LYP/6-31+G(d,p) using the C-PCM solvent model for polar organic solvents available in SPARTAN′24.

The theoretical data are in agreement with previous findings,^26,37^ as the most stable conformation of the amide dihedral angle (O=C–N–X) for **4** and **5** was determined to be antiperiplanar. *N*-methylation resulted in a significant conformational shift for compounds **6** and **7**, for which the most stable conformation was synperiplanar (Figure 2 and Table 1).

**Table 1:**
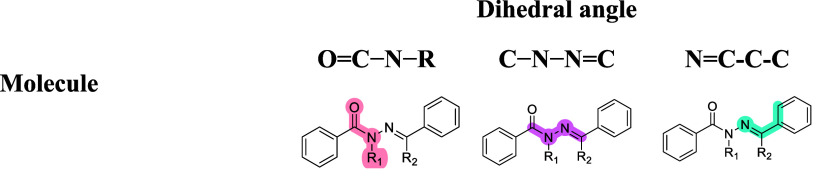

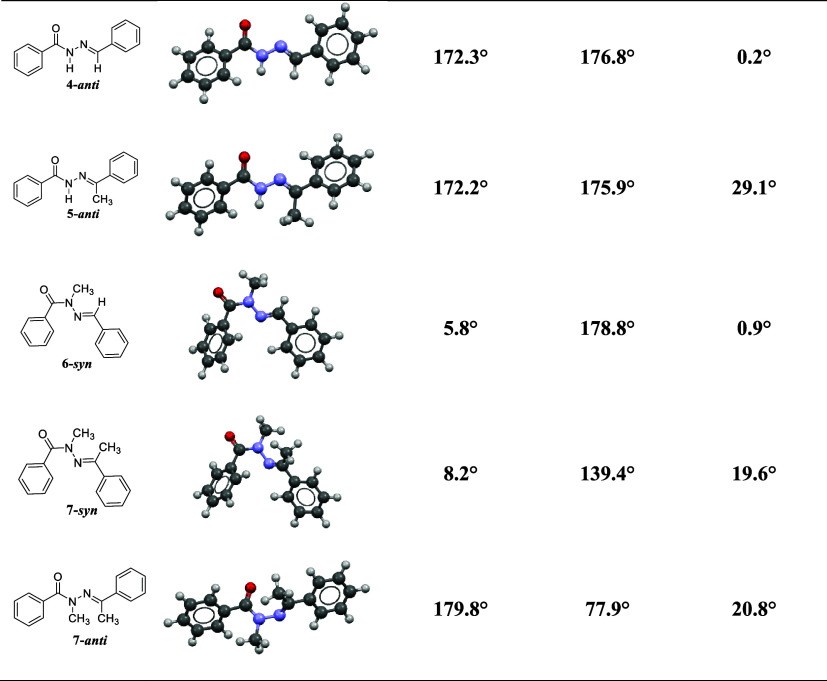
Dihedral Angles Related to the NAH Moiety of the Lowest Energy Conformers of the Analyzed Compounds (**4**–**7**)^a^

a The final geometries were obtained through equilibrium geometry calculations using the CAM-B3LYP/6-31+G(d,p) level of theory with the C-PCM solvent model for polar organic solvents available in SPARTAN′24, starting from the lowest energy conformers of the potential energy surfaces.

Notably, for compound **7**, the energy difference between ***syn*** and ***anti*** conformations was smaller compared to that of compound **6**. A Boltzmann distribution analysis at 298.15 K of the potential energy surface scan indicated that the most stable conformers of **4**, **5**, **6**, and **7** represent 77.5, 78.6, 92.8, and 68.5% of the analyzed conformers, respectively.

From the ***syn*** and ***anti*** conformers of each compound (**4**–**7**) (Figure 2), complete geometry optimizations were performed using the C-PCM solvent model for polar organic solvents in SPARTAN′24 with the CAM-B3LYP31/6-31+G(d,p) method. These calculations revealed that **4**-***anti*** is 1.71 kcal/mol more stable than **4**-***syn***, and **5**-***anti*** is also 1.71 kcal/mol more stable than **5**-***syn***. This indicates that the presence of the methyl group on the imine carbon does not significantly influence the stabilization of a different conformation of the NAH scaffold. However, a difference in the coplanarization of the ring adjacent to the imine was observed, where **5**-***anti*** showed a deviation of 29° compared to **4**-***anti***, which may potentially impact the π-electron conjugation of this system (Table 1). Similarly, a deviation of around 20° was also observed for both the ***syn*** and ***anti*** conformers of compound **7** (Table 1). As expected, compound **6** showed a significant preference for the ***syn*** conformation with an energy difference of 2.67 kcal/mol in favor of this conformer, corroborating previous studies. For compound **7**, the data indicated a slight preference for the ***syn*** conformer with a difference of 0.78 kcal/mol. Given this small energy gap between **7**-***syn*** and **7**-***anti***, both conformers were considered for further analysis.

As expected for compounds **4**, **5**, and **6**, the analysis of the final O=C–N–X and C–N–N=C dihedral angles (Table 1) indicated that, regardless of the conformation at the amide function, the NAH moiety remains planar, i.e., both the amide and imine bonds tend to lie within the same plane. This planarity is likely attributed to conjugation of the amide nitrogen lone pair with the carbonyl and imine groups.

However, the same behavior is not observed for **7**: the amide and imine bonds show a greater deviation from planarity (Table 1), suggesting that a planar conformation for the NAH group is not favorable in this case. This phenomenon is likely related to the steric effect caused by the spatial proximity of the two methyl groups in compound **7**, which disrupts the conjugation of the amide nitrogen lone pair with both the carbonyl and imine groups, as discussed below.

**Figure 3:**
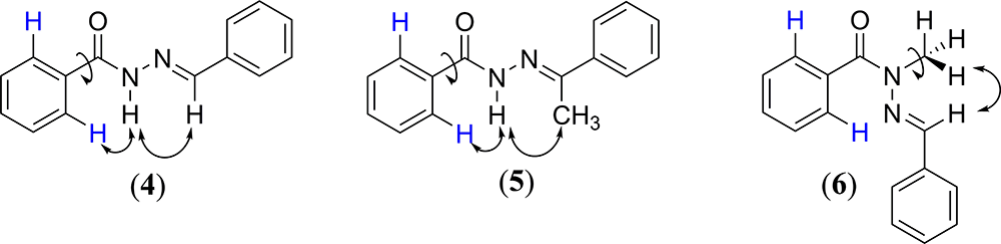
Spatial correlation between the hydrogens of the NAH moiety of the compounds (**4**–**6**).

The theoretical conformational analysis was supported by 2D NOESY (^1^H–^1^H) experiments, which revealed experimental spatial correlations between the hydrogens. Considering that the theoretical analysis indicated that the lowest energy conformers of compounds **4** and **5** have an O=C–N–X dihedral angle in the antiperiplanar conformation, spatial interactions between the amide and aromatic hydrogens would be expected and were indeed observed (Supporting Information Figures S5 and S10). Additionally, we anticipated spatial interactions between the amide hydrogens and the imine hydrogen in compound **4**, as well as between the amide hydrogens and the methyl hydrogens in compound **5**, which were confirmed (Figure 3). The lowest energy conformer of compound **6** exhibits the O=C–N–X dihedral angle in the synperiplanar conformation, consistent with the lack of spatial interactions between the amide methyl hydrogens and aromatic hydrogens (Figure 3) (Supporting Information Figures S15 and S20). Additionally, the 2D NOESY (^1^H–^1^H) analysis for compound **7** did not show any spatial interaction, which precluded the assignment of the preferred conformation in the solution.

Natural bond orbital (NBO)^38^ charges were calculated to evaluate the electron density distribution of the carbonyl and imine carbons and to correlate these with the chemical shifts observed in ^13^C NMR (Table 2). In addition, carbon chemical shift calculations were performed for compounds **4**–**7** (Table 2). The NBO and NMR analyses were conducted on the lowest energy conformers identified through conformational analysis and geometry optimization (Table 1), using CAM-B3LYP/^31^6-31+G(d,p), and employing the C-PCM/DMSO solvent model^32,33^ available in GAUSSIAN′09.

**Table 2:**
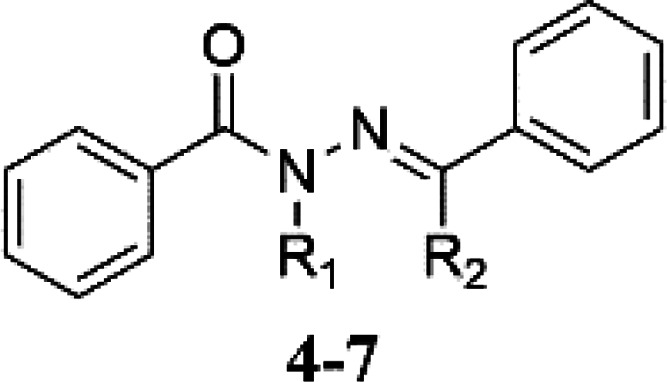
Experimental and Calculated ^13^C Chemical Shifts, Calculated NBO Charges for the Carbonyl and Imine Carbons, and NBO Analyses Performed in GAUSSIAN′09RR

		Molecule				
		**4**	**5**	**6**	**7**	
		_1_**,** R_2_ = H	R_1_ = H, R_2_ = CH_3_	R_1_ = CH_3_, R_2_ = H	_1_**,** R_2_ = CH_3_	
Entry					synperiplanar conformer	antiperiplanar conformer
*1*	^13^C δ (C=O) (ppm)	163.2	163.9	170.0	169.4	
*2*	^13^C δ (C=N) (ppm)	147.8	155.5	140.4	169.5	
*3*	calc. ^13^C δ (C=O) (ppm)^a^	168.3	168.4	177.5	178.5	167.4
*4*	calc. ^13^C δ (C=N) (ppm)^a^	150.8	157.7	143.8	176.7	181.1
*5*	C NBO charge (C=O)	0.692	0.695	0.716	0.706	0.688
*6*	C NBO charge (C=N)	0.066	0.262	0.041	0.314	0.335
*7*	*n*_N_ → π*_C=O_ (kcal/mol)	70.97	71.21	60.17	42.41	46.24
*8*	*n*_N_ → π*_C=N_ (kcal/mol)	28.93	28.85	34.68	6.54	2.36
*9*	*n*_O_ → σ*_C–C_ (kcal/mol)	21.10	21.13	20.07	20.04	20.03
*10*	*n*_O_ → σ*_C–N_ (kcal/mol)	30.34	30.22	28.48	27.29	27.98
*11*	*n*_N_ → σ*_C–R_ (kcal/mol)	11.39	12.74	12.31	13.19	13.09
*12*	π_C=C_ → π*_C=N_ (kcal/mol)	22.20	15.54	21.34	18.19	17.92
*13*	π_C=N_ → π*_C=C_ (kcal/mol)	9.07	7.19	9.20	7.30	6.95
*14*	π_C=C_ → π*_C=O_ (kcal/mol)	20.63	20.47	15.17	15.29	11.05
*15*	π_C=O_ → π*_C=C_ (kcal/mol)	3.16	3.20	2.33	2.27	1.55

a Calculated using CAM-B3LYP/^31^6-31+G(d,p), with the C-PCM/DMSO solvent model^32,33^ employing the GIAO method for NMR calculation available in GAUSSIAN′09. TMS HF/6-31G(d) GIAO was used as reference for data visualization and analyses in GaussView 5.0.9.

The experimental ^13^C chemical shift for the carbonyl group showed changes due to *N*-methylation, as observed for compounds **6** and **7**. The introduction of methyl groups into the NAH moiety affects the conjugation of this system through conformational effects, thereby influencing the electronic density of the NAH carbon atoms. For example, compared to compound **4**, the imine carbon chemical shift of **5** is 7.7 ppm higher. In contrast, the same carbon of **6** shows a chemical shift 7.4 ppm lower, while the carbonyl carbon’s chemical shift is 6.8 ppm higher. After the *N*-methylation, the carbonyl carbon becomes more electron-deficient, as indicated by the increased positive NBO charges for the carbonyl carbons in compound **6** and the synperiplanar conformer of **7**. In compound **7**, the characteristic steric effect of the two methyl groups reduces NAH planarity, disrupting the conjugation, which is reflected in the chemical shifts of both the carbonyl and imine carbons compared to compound **4**. Moreover, the chemical shift values calculated using the GIAO method^39^ are in agreement with the experimental data. The comparison between the observed and predicted values may shed some light on the preferred conformation of compound **7** in solution. To this end, we analyzed the complete data set for all compounds, considering either the **7**-***syn*** or the **7**-***anti*** conformer. The *R*^2^ values using **7**-***syn*** were 0.9936, while for **7**-***anti***, they were 0.9546. The root mean square error (RMSE) values were 5.72 and 5.77, respectively. These results point to **7**-***syn*** as the preferred conformer in solution.

According to NBO data, there are differences in the conjugation of the lone pair of the amide nitrogen of the NAH moiety with the π* orbitals of the C=O and N=C double bonds in each system (Table 2). Using compound **4** as a reference, the presence of a methyl group at the imine carbon of compound **5** did not significantly affect the electronic donation from the *n*_N_ orbital to the π*_C=O_ and π*_C=N_ orbitals (entries 9 and 10, Table 2), indicating no notable change in the conjugation of the NAH moiety (Table 2). Consequently, the system should remain planar, as later confirmed by the X-ray data. The greater chemical shift related to the imine carbon of **5** in comparison to **4** (entries 2 and 4, Table 2) can be explained by a deeper look into the NBO analysis. The methylation at the imine carbon decreased the resonance of the imine group with the neighboring phenyl ring (entries 12 and 13, Table 2). Regarding compound **6**, the reduced donation of the amide nitrogen lone pair (*n*_N_) to the π*_C=O_ by ∼10 kcal/mol and increased donation of the *n*_N_ to the π*_C=N_ by ∼6 kcal/mol resulted in a higher charge of the carbonyl carbon and a corresponding increase in its chemical shift, while the imine carbon was shielded (entries 1–6, Table 2). As expected, the conjugation in NAH derivative **7**, irrespective of the conformation analyzed, was the most affected compared to compound **4**. Significant conformational effects caused by high steric hindrance led to a strong decrease of conjugation of the *n*_N_ orbital with the π*_C=O_ and π*_C=N_ orbitals, resulting in conjugation energies diminished by 28 and 22 kcal/mol for the synperiplanar conformation, and by 24 and 26 kcal/mol for the antiperiplanar conformation, respectively (entries 7 and 8, Table 2). These effects are also evidenced by the chemical shift values and NBO charges. Furthermore, an analysis of other strong interactions using second-order perturbation theory indicated that the methylation pattern does not significantly affect the hyperconjugation of the carbonyl oxygen lone pair (*n*_O_) with the adjacent σ*_C–C_ or σ*_C–N_ bonds (entries 9 and 10, Table 2). Similarly, the hyperconjugation of the imine nitrogen lone pair with the neighboring σ*_C–R_ bond of the imine carbon was not significantly altered (entry 11, Table 2). However, the resonance between the phenyl ring and the carbonyl group was significantly affected by the methylation of the amide nitrogen (entries 14 and 15, Table 2).

The second-order perturbation theory analysis aligns well with the previous NBO analysis published by our group.^35,36^ In contrast, other studies reporting NBO analysis with similar systems suggest possible intramolecular interactions through remote hyperconjugation, which may contribute to conformational stabilization,^35,40−42^ a phenomenon not observed in this study.

NBO analysis and NCI analysis^43^ are complementary approaches for understanding intramolecular and intermolecular interactions. While NBO analysis provides insight into electronic delocalization, charge transfer, and orbital interactions within a molecule, NCI analysis focuses on the visualization and characterization of interactions such as hydrogen bonding, van der Waals forces, and steric effects. By combining these methods, we aimed to gain a greater understanding of conformational preferences. The reduced density gradient (RDG) approach is a tool to visualize and quantify these interactions.

In this study, NCI analysis^43^ was performed using the Multiwfn program^44,45^ to investigate the impact of NAH methylation patterns on the nature and distribution of noncovalent interactions. The analysis was conducted for the optimized conformers of each different *N*-acylhydrazone derivative, **4**–**7**. For compound **7**, both *syn*- and *anti*-conformers, identified through conformational analysis (Table 1), were included in the NCI study.

**Figure 4:**
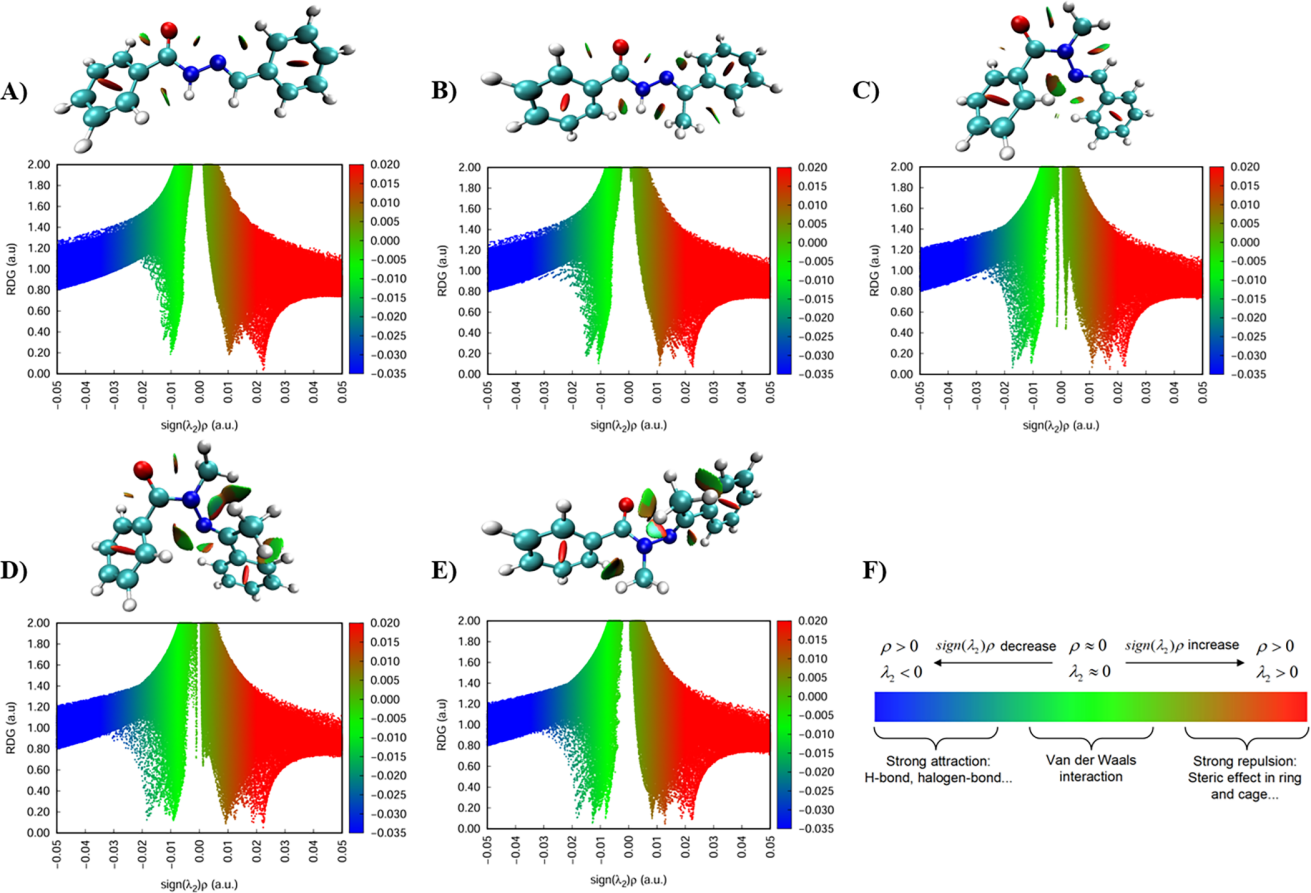
Noncovalent interaction (NCI) analysis of compounds **4**–**7**. (A–E) Molecular structures with their corresponding reduced density gradient (RDG) isosurfaces and RDG scatter plots of **4**, **5**, and **6** and **7**-***syn*** and **7**-***anti***, respectively. The isosurfaces highlight regions of noncovalent interactions, color-coded according to the interaction type: blue for strong attractive interactions (e.g., hydrogen bonding), green for weak van der Waals interactions, and red for strong steric repulsion. The RDG scatter plots display the sign(λ_2_)ρ values, where negative values correspond to attractive interactions, near-zero values indicate van der Waals forces, and positive values represent repulsion. (F) Color scale used in the analysis.

Figure 4 presents the NCI analysis of these molecular structures using the RDG approach, with the results visualized as 3D isosurfaces and 2D RDG scatter plots. Each molecular structure (Figure 4A–E) is associated with an RDG plot, where the *X*-axis and *Y*-axis correspond to sign(λ_2_)ρ and RDG functions, respectively, which helps distinguish between different interaction types. The blue regions indicate strong attractive interactions, such as hydrogen bonding or halogen bonding; the green regions represent weak van der Waals interactions; and the red regions correspond to steric repulsion effects, typically due to ring strain or congestion. The color scale (Figure 4F) provides a reference for interpreting the interaction intensities. This analysis allows for a comparative evaluation of how methylation influences NCIs, offering information on molecular stability and conformation. Values below 0.5 RDG indicate the occurrence of such noncovalent interactions and correspond to the points used to construct the 3D isosurfaces. In comparison to the nonmethylated compound **4** (Figure 4A), all others have an increased contact surface area indicating a slight increase in weak van der Waals interactions along with increased steric repulsion that goes higher as the number of methyl groups increases.

**Figure 5:**
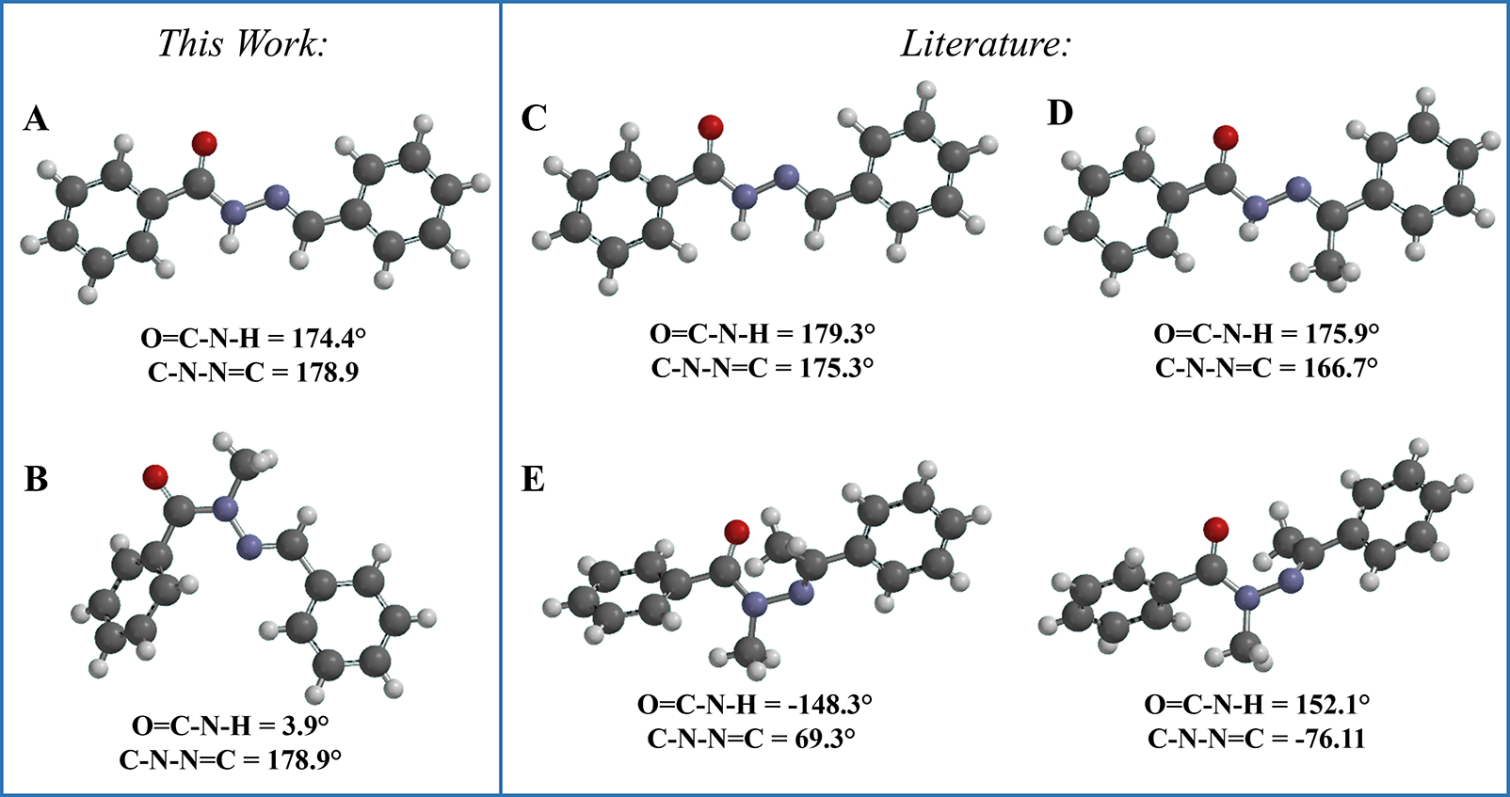
Crystal structures of compounds (**4**), (**5**), (**6**), and (**7**). (A) and (B) represent the crystal structures of compounds (**4**) and (**6**), respectively, as determined in this study. (C), (D), and (E) correspond to crystal structures of (**4**) (CCDC ID: 1287640),^55^ (**5**) (CCDC ID: 706086),^56^ and (**7**) (CCDC ID: 1006339),^57^ respectively, which are already deposited in the CCDC database.

To corroborate the theoretical and NMR conformational analyses and confirm the presence of a single diastereomer, we conducted powder X-ray diffraction analyses. Similar detailed procedures can be found elsewhere.^35,46−49^ We used the first 20 reflections of the diffraction patterns to index them. Briefly, it was possible to determine the crystal structures of compounds **4** and **6** (Figure 5) using a simulated annealing^50^ approach implemented in TOPAS-Academic v.7 software^51^ by constructing a rigid-body structure of the compounds. The final structures were refined using the Rietveld method^52,53^ (Figures S25 and S26 display the final Rietveld plots for **4** and **6** respectively, Supporting Information). Compound **4** crystallized in an orthorhombic crystal system—space group *Pna*2_1_ (nr. 33), unit cell parameters: *a* = 8.78029(16) Å, *b* = 10.45095(17) Å, *c* = 13.0923(3) Å, and *V* = 1201.38(4) Å^3^—and the relative configuration of the imine double bond (C=N) was evaluated as being *E*. The unit cell is composed of four formula units (*Z* = 4) with one molecule in the asymmetric unit (*Z*′ = 1). As expected, the molecules adopted a planar shape within the unit cell. Compound **6** crystallized in a monoclinic crystal system—space group *P*2_1_/*c* (nr. 14), unit cell parameters: *a* = 4.05267(6) Å, *b* = 11.5520(3) Å, *c* = 27.0148(7) Å, β = 92.950(2)°, and *V* = 1263.05(5) Å^3^—and the relative configuration of its imine double bond (C=N) was also *E*. The unit cell is composed of four formula units (*Z* = 4) and one molecule in the asymmetric unit (*Z*′ = 1). Within the unit cell, the molecules assumed a folded shape.

We also searched for the compounds analyzed in this work in the CCDC database with the ConQuest software.^54^ It was found that compounds **4**, **5**, and **7** were already deposited in the CCDC database under the CCDC IDs 1287640,^55^ 706086,^56^ and 1006339,^57^ respectively (Figure 5). Our crystal structure determination of compound **4** closely matches the previously deposited structure, further corroborating both our theoretical and experimental data. Compound **6**, as expected, presented a synperiplanar conformation. For compound **7**, two distinct conformations were determined within the same crystallographic structure, differing primarily in the orientation of the imine methyl group. Both conformations of **7** show the amide methyl group in an antiperiplanar conformation relative to the carbonyl oxygen, which does not fully align with our theoretical analysis.

Overall, the interactions influencing crystal packing of **7** are governed by nonclassical hydrogen bonding and π–π stacking interactions (as shown in Supporting Information, Figure S27). The unit cell data for compound **7** indicate a close proximity between phenyl rings, suggesting a T-shaped π–π stacking interaction. Additionally, nonconventional hydrogen bonds occur between polarized N–CH_3_ protons and the carbonyl oxygen, both intra- and intermolecular, as well as with the imine nitrogen. These close contacts may contribute to the stability of the solid state. However, due to the low energy of these interactions, they are unlikely to significantly affect the main conformations in the solution state, possibly explaining the observed differences compared to those in the solid state.

For the crystal structure determinations of compounds **4** and **6**, we used PLATON^58^ and Mercury^59,60^ software to verify the molecular geometry, including the correct selection of space groups, unit cell parameters, bond distances, angles, and torsions. The supplementary crystallographic data for compounds **4** and **6** have been deposited with the CCDC under IDs 1908136 and 1908137, respectively. It is also important to note that all of the analyzed compounds exhibited the single diastereomer *E*.

**Figure 6:**
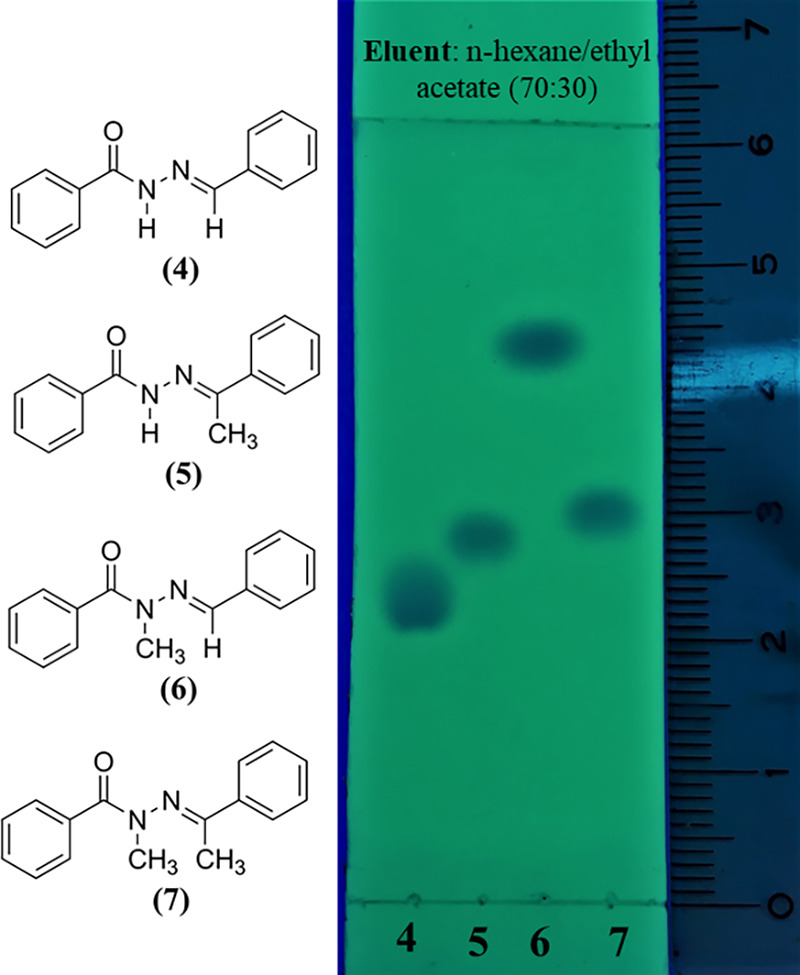
Thin-layer chromatography (TLC) for compounds **4**–**7**. TLC was performed on 2.0 × 6.0 cm aluminum sheets precoated with silica gel 60 (HF-254, Merck) to a thickness of 0.25 mm, using *n*-hexane/ethyl acetate (70:30) as the solvent system. The spots were visualized under ultraviolet light at 254 nm.

The impact of *N*-methylation on the NAH moiety’s polarity is evident when analyzing both the thin-layer chromatography (TLC) retention factors (*R*_f_) and the melting points of the compounds (Figure 6 and Table 3). The introduction of a methyl group typically reduces the polarity, which is reflected in the *R*_f_ values and corroborated by the dipole moments, polar surface area (PSA), and cLogP values from Table 3.

**Table 3:** *N*-Methylation Impacts Polarity in the NAH Moiety

molecule	*R*_f_	melting point (°C)	dipole moment (D)	PSA (Å^2^)	cLogP
**4**	0.35	208–211	6.93	33.5	3.36
**5**	0.45	148–151	7.41	31.4	2.93
**6**	0.71	79–80	4.71	21.2	3.60
**7**-***syn***	0.48	108–110	6.41	20.9	3.16
**7**-***anti***			4.08	20.9	3.16

For compound **4**, the absence of *N*-methylation results in an *R*_f_ of 0.35, a melting point of 208–211 °C, and a dipole moment of 6.93 D and cLogP of 3.3. Upon *C-*methylation to form **5**, the *R*_f_ increases to 0.45, consistent with decreased polarity, while the melting point drops to 148–151 °C. The reduction in PSA (from 33.5 to 31.4 Å^2^) and the slight increase in the dipole moment (7.41 D) and decrease in cLogP highlight the complex interplay between electronic and steric effects of the *C*-methyl group.

The *N*-methylation in **6** removes a hydrogen bond donor, significantly reducing PSA to 21.2 Å^2^ and dipole moment to 4.71 D while increasing cLogP, leading to an *R*_f_ of 0.71 and a much lower melting point (79–80 °C). This pronounced reduction in polarity aligns with the expectations.

Interestingly, the comparison between compounds **5** and **7** reveals a subtler effect. While **7** has two methyl groups, its *R*_f_ (0.48) is only slightly higher than that of **5**, suggesting a limited reduction in polarity. This observation is supported by the relatively small difference in PSA values (31.4 Å^2^ for **5** and 20.9 Å^2^ for **7**) and dipole moments (7.41 D for **5** and 6.41 D for **7-*syn***). The unexpected similarity in polarity between **5** and **7** may be attributed to conformational effects, as suggested in the text. The dipole moment of **7-*anti***, at 4.08 D, is significantly lower than that of **7-*syn***, highlighting the role of the conformation in modulating molecular properties. This conformational variability is further reflected in the melting points, with **7** exhibiting intermediate values (108–110 °C) compared to **6** and **5**.

## Conclusions

The conformational effects of *N*- and *C*-methylated *N*-acylhydrazone derivatives **4**–**7** were investigated. The compounds were synthesized in suitable yields and structurally characterized by ^13^C and ^1^H NMR. Electronic differences, as reflected in the chemical shifts of carbonyl and imine carbons, were corroborated by electron density and conjugation effects through NBO calculations. The presence of two methyl groups reduced the planarity of the NAH subunit, leading to the disruption of conjugation and resulting in significant changes in chemical shifts and increased NBO charges.

The significance of the conformational effects caused by NAH methylation was further evaluated through theoretical calculations, including determination of potential energy curves, which revealed substantial conformational changes due to *N*-methylation. The impact of *N*-methylation on the polarity of the compounds was also examined by TLC. Interestingly, the compound with two methyl groups was found to be more polar than the one with just a single methyl group on the amide nitrogen atom.

The investigation into the conformational effects of *N*- and *C*-methylated *N*-acylhydrazones is highly significant, as conformational dynamics and intramolecular interactions are critical for the design and optimization of drug-like molecules. For drug-like molecules, conformational studies are recognized as an effective medicinal chemistry strategy and have served as a key optimization step for drugs containing similar functional groups, such as ureas.^61,62^

The findings from this study offer valuable insights into the physicochemical properties of *N*-acylhydrazone derivatives modified at positions R_1_ and R_2_. These results provide practical guidance for the application of this versatile class of compounds in diverse areas of chemistry and medicinal chemistry, advancing their potential utility in drug development and other related fields.

## Experimental Section

### General Information

The melting points of compounds (**4**–**7**) were determined using a Quimis 340 apparatus and are uncorrected. ^1^H NMR spectra were determined in dimethyl sulfoxide-*d*_6_ containing approximately 1% tetramethylsilane (TMS) as an internal standard using a Bruker AVANCE 500 instrument at 500 MHz. ^13^C NMR spectra were resolved using the same spectrometer at 125 MHz and exploited with the same solvent. The NMR experiments were performed with 50 mg/mL of the tested compounds in DMSO-*d*_*6*_, and chemical shifts (δ) are expressed in parts per million (ppm). IR spectra (cm^–1^) were obtained using a Thermo Scientific Nicolet Module Smart ITR. The progress of all reactions was monitored through thin-layer chromatography performed on 2.0 × 6.0 cm^2^ aluminum sheets precoated with silica gel 60 (HF-254, Merck) to a thickness of 0.25 mm. The developed chromatograms were viewed under ultraviolet light (254–366 nm) and treated with iodine vapor. The reagents and solvents were purchased from commercial suppliers and used as received. The high-resolution mass spectrometry (Orbitrap-HRMS) analysis was performed using a QExactive Hybrid Quadrupole Orbitrap Mass Spectrometer (Thermo Fisher Scientific, Waltham, USA) and electrospray ionization (ESI). Standards working solutions of the compounds (1 μg/mL) were prepared with water/methanol 7:3, fortified with 0.1% formic acid and 5 mM NH_4_COOH (ammonium formate).

#### General Procedure for the Preparation of (*E*)-*N*′-Benzylidenebenzohydrazide (4) and (*E*)-*N′*-(1-Phenylethylidene)benzohydrazide (**5**)

In a microwave flask (Monowave 300; G30 type), benzohydrazide (**1**) (0.5 g, 3.6 mmol), benzaldehyde (**2**) or acetophenone (**3**) (3.6 mmol), 10 mL of ethanol, and one drop of acetic acid, which was used as the catalyst, were added. The Monowave 300 microwave was programmed to reach 80 °C in 2 min, and the reaction was maintained under microwave irradiation for 30 min at 80 °C. Extensive precipitation was observed, and the white solid was filtered under a vacuum.

#### General Procedure for the Preparation of (*E*)-*N*′-Benzylidenebenzohydrazide (4) and (*E*)-*N′*-(1-Phenylethylidene)benzohydrazide (**5**)

In a microwave flask (Monowave 300; G30 type), benzohydrazide (**1**) (0.5 g, 3.6 mmol), benzaldehyde (**2**) or acetophenone (**3**) (3.6 mmol), 10 mL of ethanol, and one drop of acetic acid, which was used as the catalyst, were added. The Monowave 300 microwave was programmed to reach 80 °C in 2 min, and the reaction was maintained under microwave irradiation for 30 min at 80 °C. Extensive precipitation was observed, and the white solid was filtered under a vacuum.

#### (*E*)-*N*′-Benzylidenebenzohydrazide (**4**)

The title compound was obtained as a white powder at 82% yield; mp 208–211 °C (lit [46] 208–209 °C). ^1^H NMR (500 MHz, DMSO-*d*_6_) δ 11.89 (br s, 1H), 8.49 (br s, 1H), 7.95 (d, 2H, *J* = 7.4 Hz), 7.75 (d, 2H, *J* = 7.0 Hz), 7.61 (dd, 1H, *J* = 8.3 and 7.4 Hz), 7.54 (dd, 2H, *J* = 8.3 and 7.4 Hz), 7.50–7.42 (m, 3H). ^13^C NMR (125 MHz, DMSO-*d*_6_) δ: 163.2, 147.8, 134.3, 133.4, 131.7, 130.1, 128.8, 128.5, 127.6, 127.1. IR (ATR, cm^–1^): 3176, 1638, 1601, 1552, 1362, 1284. HRMS calculated for C_14_H_13_N_2_O: [M + H]^+^ = 225.10279 found: *m*/*z* 225.10396.

#### (*E*)-*N′*-(1-Phenylethylidene)benzohydrazide (**5**)

The title compound was obtained as a white powder at 62% yield; mp 148–151 °C (lit [46] 155–156 °C). ^1^H NMR (500 MHz, DMSO-*d*_6_) δ 10.79 (br s, 1H), 7.95–7.75 (m, 4H), 7.58 (dd, 1H, *J* = 8.2 and 7.3 Hz), 7.52 (dd, 2H, *J* = 8.2 and 7.3 Hz), 7.48–7.37 (m, 3H), 2.38 (s, 3H). ^13^C NMR (125 MHz, DMSO-*d*_6_) δ: 163.9, 155.5, 138.1, 134.1, 131.5, 129.4, 128.3, 128.3, 127.9, 126.4, 14.6. IR (ATR, cm^–1^): 3168, 1653, 1636, 1602, 1538, 1281. HRMS calculated for C_15_H_15_N_2_O: [M + H]^+^ = 239.11844 found: *m*/*z* 239.11779

#### General Procedure for the Preparation of (*E*)-*N*′-Benzylidene-*N*-methylbenzohydrazide (**6**) and (*E*)-*N*-Methyl-*N′*-(1-phenylethylidene)benzohydrazide (**7**)

A solution of (*E*)-*N′*-benzylidenebenzohydrazide (**4**) or (*E*)-*N′*-(1-phenylethylidene)benzohydrazide (**5**) (1.33 mmol) and potassium carbonate (0.554 g, 4.0 mmol) were suspended in 20 mL of acetone in a round-bottom flask. The suspension was thoroughly mixed under vigorous stirring for 5 min, and methyl iodide (0.416 mL, 6.685 mmol) was subsequently added. The reaction mixture was heated at 50 °C and maintained under stirring for 18 h. Subsequently, the reaction mixture was partially evaporated under reduced pressure and the residual mixture was poured into cold water. The solid was collected through filtration. The solid was recrystallized in EtOH/H_2_O.

#### (*E*)-*N*′-Benzylidene-*N*-methylbenzohydrazide (**6**)

The title compound was obtained as a white powder at 80% yield; mp 79–80 °C (lit. [47] 84 °C). ^1^H NMR (500 MHz, DMSO-*d*_6_) δ 8.03 (br s, 1H), 7.64 (d, 2H, *J* = 7.2 Hz), 7.54–7.49 (m, 3H), 7.47 (dd, 2H, *J* = 8.3 and 7.2 Hz), 7.40–7.33 (m, 3H), 3.51 (s, 3H). ^13^C NMR (125 MHz, DMSO-*d*_6_) δ 170.0, 140.4, 135.5, 134.8, 130.0, 129.4, 129.3, 128.7, 127.4, 126.9, 28.7. IR (ATR, cm^–1^): 1647, 1608, 1603, 1456, 1404, 1346, 1051. HRMS calculated for C_15_H_15_N_2_O: [M + H]^+^ = 239.11844 found: *m*/*z* 239.12041

#### (*E*)-*N*-Methyl-*N′*-(1-phenylethylidene)benzohydrazide (**7**)

The title compound was obtained as a white powder at 37% yield; mp 108–110 °C. ^1^H NMR (500 MHz, DMSO-*d*_6_) δ 7.91–7.27 (m, 10H), 3.27 (s, 3H), 2.31 (s, 3H). ^13^C NMR (125 MHz, DMSO-*d*_6_) δ: 169.5, 169.4, 136.9, 135.7, 130.6, 130.0, 128.4, 127.9, 127.9, 126.9, 36.1, 16.9. IR (ATR, cm^–1^): 1627, 1573, 1445, 1340, 1302, 1054. HRMS calculated for C_16_H_17_N_2_O: [M + H]^+^ = 253.13409 found: *m*/*z* 253.13416.

### X-ray Powder Diffraction

The samples were gently hand-ground in an agate mortar and then loaded between two acetate/cellulose foils (0.014 mm thick) in a sample holder held spinning during data collection. The data were recorded at room temperature (295 K) on a STADI-P powder diffractometer from Stoe (Darmstadt, Germany) in transmission geometry by using Cu *K*α_1_ radiation (λ = 1.54056 Å) and selected by a curved monochromator Ge (111), with a tube voltage of 40 kV and a current of 40 mA. The intensities were collected by a silicon microstrip detector, Mythen 1K (Dectris, Baden, Switzerland). The ranges used for the data collection were from 9 to 71.985° to the compound **4** sample and from 2 to 90.185° to the compound **6** sample with steps of 0.015° and a counting time of 200 s at each 1.05°.

### Molecular Modeling

Potential energy surface (PES) scan analyses and equilibrium geometry calculations were performed with Spartan’24 software (Wavefunction, Inc.). NMR calculations (using the GIAO method^39^) and NBO^38^ analysis were performed using the GAUSSIAN’09 (Revision-D.01) software package. All theoretical calculations were performed with the CAM-B3LYP density functional^31^ level of theory using the 6-31+G(d,p) basis set with the C-PCM solvent model^32,33^ for polar organic solvent (ε = 37.22) option available in Spartan’24 software or C-PCM/DMSO when the calculation was performed in GAUSSIAN’09 (Revision-D.01). The PES scans were calculated in a relaxed manner. Dipole moment, PSA, and cLogP properties were calculated in Spartan’24 software (Wavefunction, Inc.). The NCI analysis was performed using the Multiwfn program.^44,45^ Wave function (wfn) files were generated after the NBO analysis calculations, using the same level of theory. 3D isosurfaces were visualized using the VMD program following the instructions provided in the Multiwfn manual. 2D RDG scatter plots were generated using Gnuplot (http://www.gnuplot.info/), as described in the Multiwfn manual.

## Data Availability

The data supporting this article have been included as part of the Supporting Information. Crystallographic data for compounds **4** and **6** has been deposited at the CCDC under the IDs 1908136 and 1908137, respectively.

## Supporting Information Available

Experimental methods and spectroscopic data for the studied compounds; general information on the determination of melting points, NMR (^1^H and ^13^C) spectroscopy, infrared spectroscopy, and reaction monitoring by thin-layer chromatography (TLC), along with details on the reagents and solvents used; NMR spectral data for compounds **4**–**7** (Figures S1–S20) and high-resolution mass spectrometry (HRMS) data for the same compounds (Figures S21–S24); final Rietveld refinement plots for compounds **4** and **6** (Figures S25 and S26) and crystal packing interactions for compound **7** (Figure S27); and comprehensive structural and spectroscopic characterization of the synthesized compounds (PDF)
