# Supplementary material for: Exploring the Conformational Effects of N- and C-Methylation of N-Acylhydrazones
Source: ACS Omega. 2025 Apr 21;10(17):17993–8004. doi: 10.1021/acsomega.5c01289 (PMC12059928; doi:10.1021/acsomega.5c01289)
Supplement: Supplementary file 1 — ao5c01289_si_001.pdf [file ao5c01289_si_001.pdf]

Supporting Information

Exploring the Conformational Effects of *N*- and *C*-Methylation of *N*-acylhydrazones

Lucas Silva Franco,[a,b] Marina Amaral Alves,[b,c] Carlos Mauricio R. Sant'Anna,[a,b,d] Isadora Tairinne de Sena Bastos,[h] Regina Cely Rodrigues Barroso,[e] Fanny Nascimento Costa,[f] Fabio Furlan Ferreira,[f] Carlos A. M. Fraga,[†,a,b] Eliezer J. Barreiro,[†,a,b] Lídia Moreira Lima,[a,b] Daniel A. Rodrigues,[*,g] and Pedro de Sena M. Pinheiro[*,a,b]

[a.] Laboratório de Avaliação e Síntese de Substâncias Bioativas (LASSBio), Instituto de Ciências Biomédicas, Universidade Federal do Rio de Janeiro, 21941-902, Rio de Janeiro, RJ, Brazil

[b.] Instituto Nacional de Ciência e Tecnologia de Fármacos e Medicamentos (INCT-INOFAR), CCS, Universidade Federal do Rio de Janeiro, Cidade Universitária, Rio de Janeiro 21941-902, RJ, Brazil

[c.] Walter Mors Institute of Research on Natural Products, Federal University of Rio de Janeiro (UFRJ), Rio de Janeiro, RJ, Brazil

[d.] Departamento de Química Fundamental, Instituto de Química, Universidade Federal Rural do Rio de Janeiro, 23970-000, Seropédica, RJ, Brazil

[e.] LabFisMed, State University of Rio de Janeiro, Physics Institute, Rio de Janeiro 20550-900, RJ, Brazil

[f.] Center for Natural and Human Sciences, Federal University of ABC, Santo André 09210-580, SP, Brazil

[g.] School of Pharmacy and Biomolecular Sciences (PBS), Royal College of Surgeons in Ireland, 1st Floor Ardilaun House Block B 111 St Stephen's Green, Dublin 2, Ireland

[h.]Department of Physics, State University of Feira de Santana, 44036-900, Feira de Santana, BA, Brazil

[†] In memoriam

[*]To whom correspondence should be addressed. E-mail: danielalencar@rcsi.com; pedro.pinheiro@icb.ufrj.br

Contents of SI:

- General Information.
- NMR spectral data of the compounds **4** – **7** – **Figure S1 – Figure S20**.
- High-resolution mass spectrometry of the compounds **4** – **7** – **Figure S21 – Figure S24**.
- Final Rietveld plots for compounds **4** and **6** – **Figures S25** and **S26**.
- Crystal packing contacts for compound **7** – **Figure S27**.

General information. The melting points of **4-7** were determined using a Quimis 340 apparatus and are uncorrected. ^{1}H-NMR spectra were determined in dimethyl sulfoxide containing approximately 1% tetramethylsilane (TMS) as an internal standard using a Bruker AVANCE 500 at 500 MHz. ^{13}C-NMR spectra were resolved using the same spectrometer at 125 MHz and exploited the same solvent. The NMR experiments were performed with 50 mg/mL of the tested compounds in DMSO-d_6. Chemical shifts (δ) are expressed in parts per million (ppm). IR spectra (cm^{-1}) were obtained using a Thermo Scientific Nicolet module Smart ITR. The progress of all the reactions was monitored through thin-layer chromatography performed on 2.0 × 6.0-cm^2 aluminum sheets precoated with silica gel 60 (HF- 254, Merck) to a thickness of 0.25 mm. The developed chromatograms were viewed under ultraviolet light (254−366 nm) and treated with iodine vapor. The reagents and solvents were purchased from commercial suppliers and used as received. The high-resolution mass spectrometry (Orbitrap-HRMS) analysis was performed using a QExactive Hybrid Quadrupole Orbitrap Mass Spectrometer (Thermo Fisher Scientific, Waltham, USA) using electrospray ionization (ESI). Standards working solutions of the compounds (1µg/mL) were prepared with water/methanol 7:3, fortified with 0.1% formic acid and 5mM NH_4COOH (ammonium formate).

Technical assistance was provided by Camila B. O. Mansur and Francisco A. V. dos Santos from Laboratório Multiusuário de Análises por RMN (LAMAR) of Instituto de Pesquisas de Produtos Naturais Walter Mors (Universidade Federal do Rio de Janeiro), and Roberta L. C. Trindade from Laboratório de Ressonância Magnética Nuclear em Solução (LABRMN-2) of Instituto de Química (Universidade Federal do Rio de Janeiro).

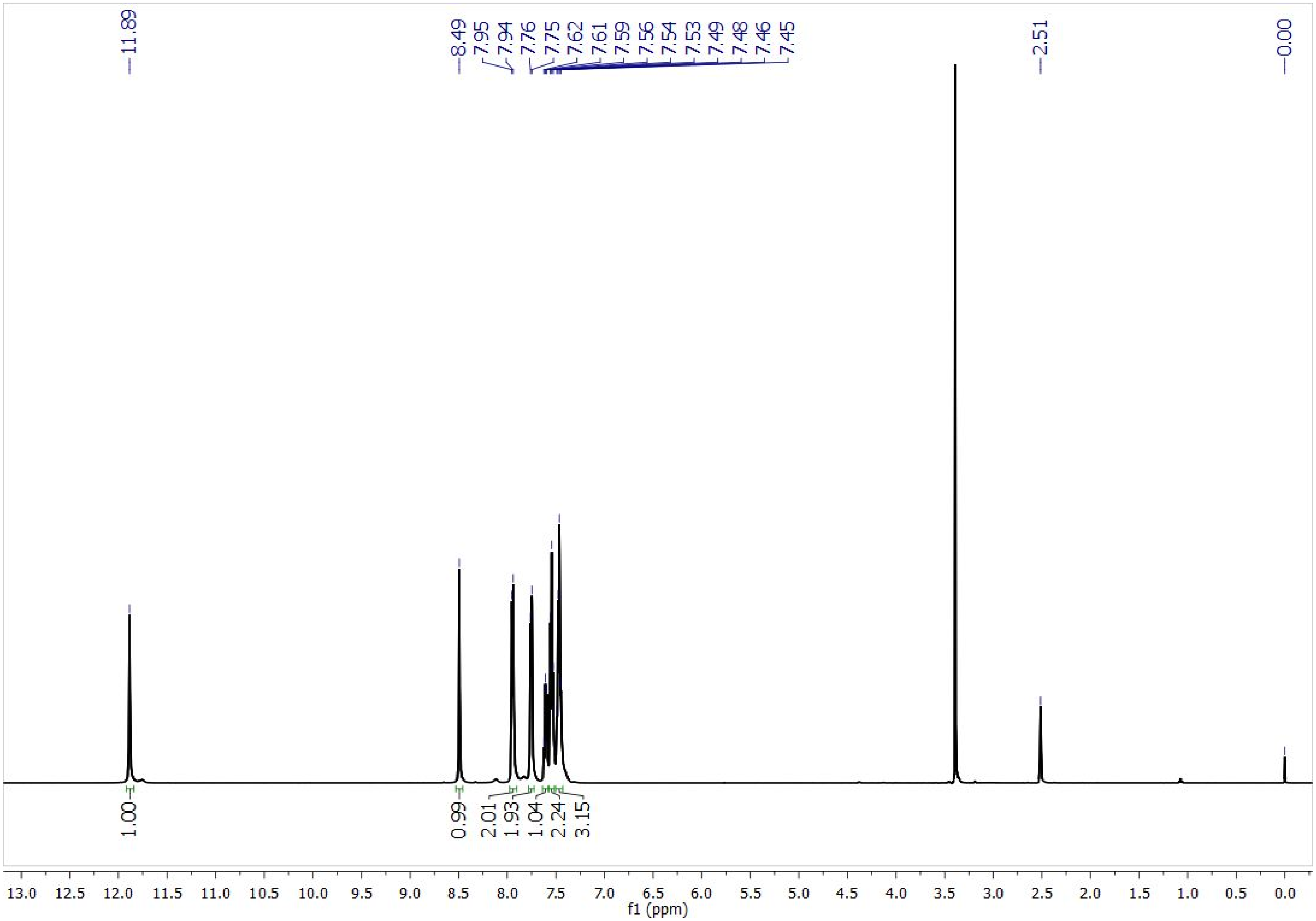

Figure S1 – Compound **4** (^{1}H NMR, 500 MHz, DMSO-d_6, TMS).

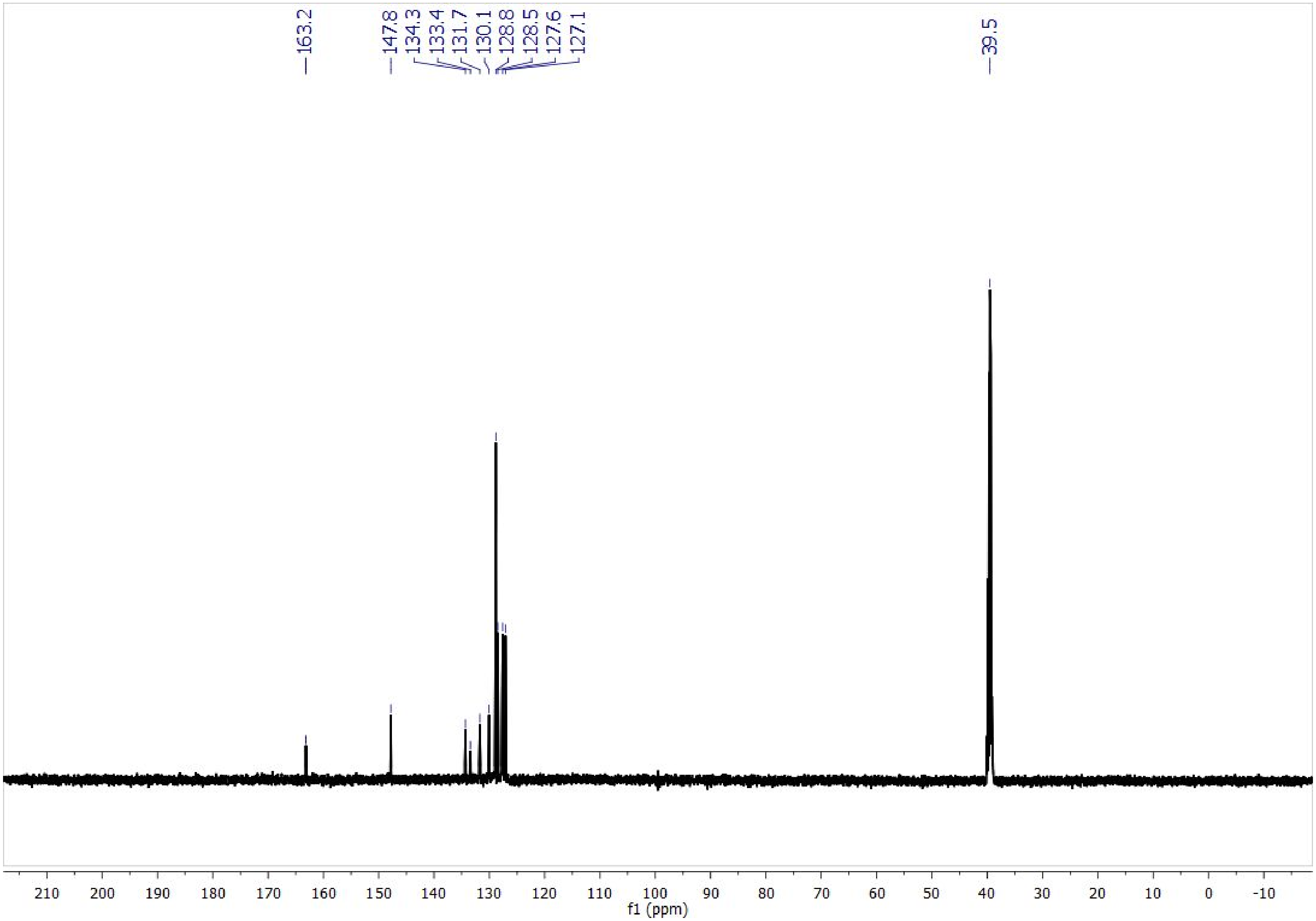

Figure S2 – Compound **4** (^{13}C NMR, 125 MHz, DMSO-d_6, TMS).

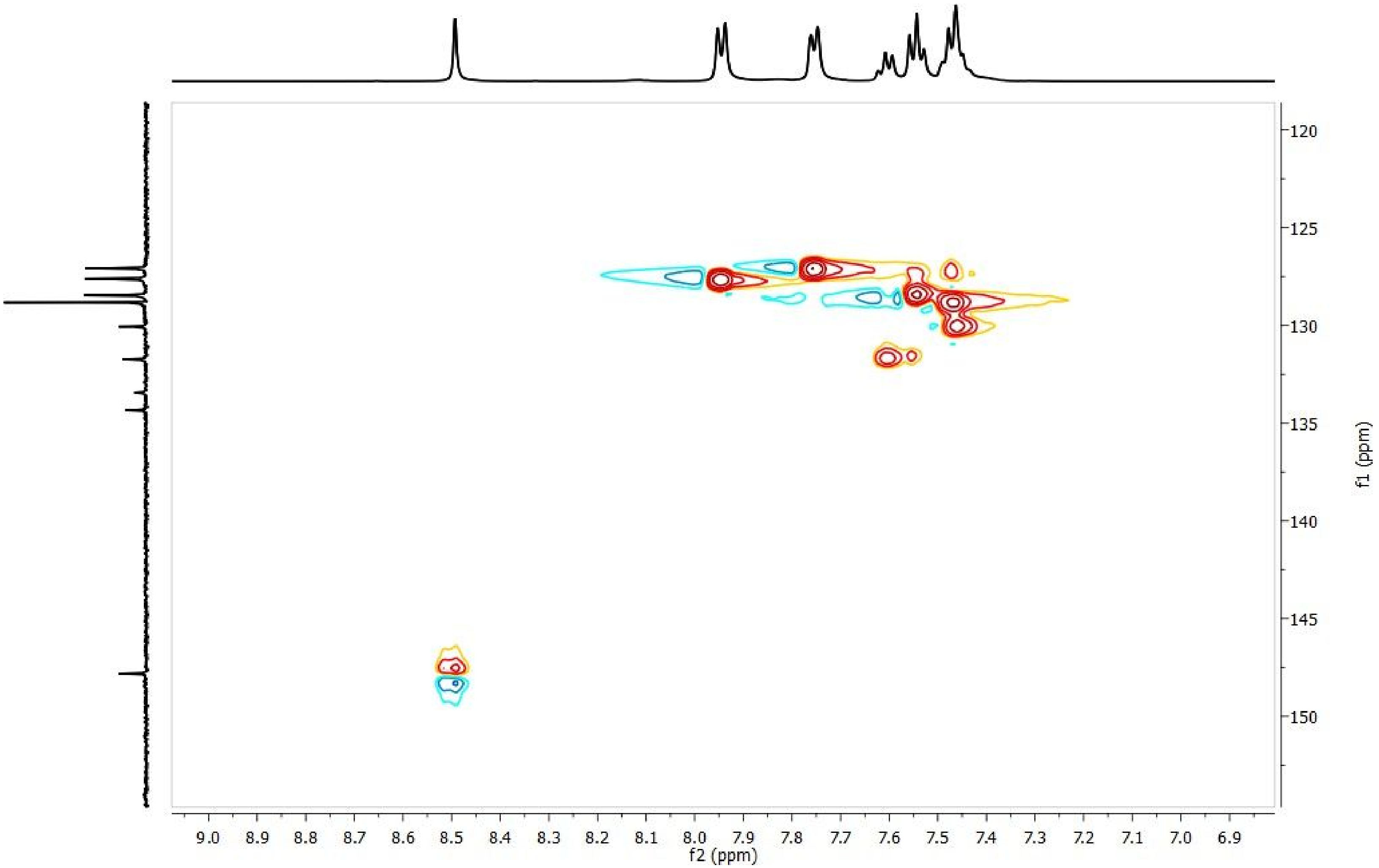

Figure S3 – 2D NMR HSQC spectrum of compound **4.**

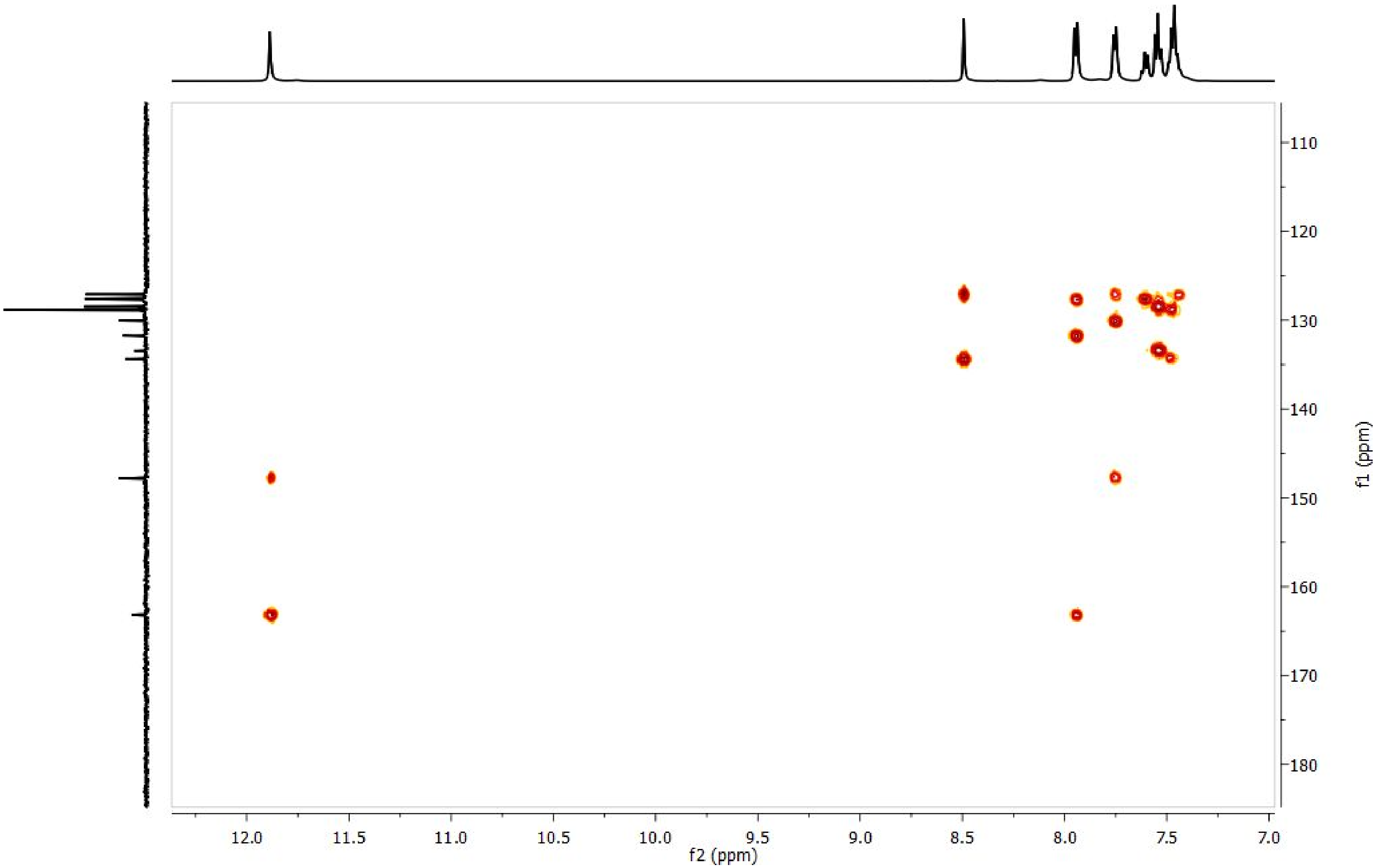

Figure S4 – 2D NMR HMBC spectrum of compound **4.**

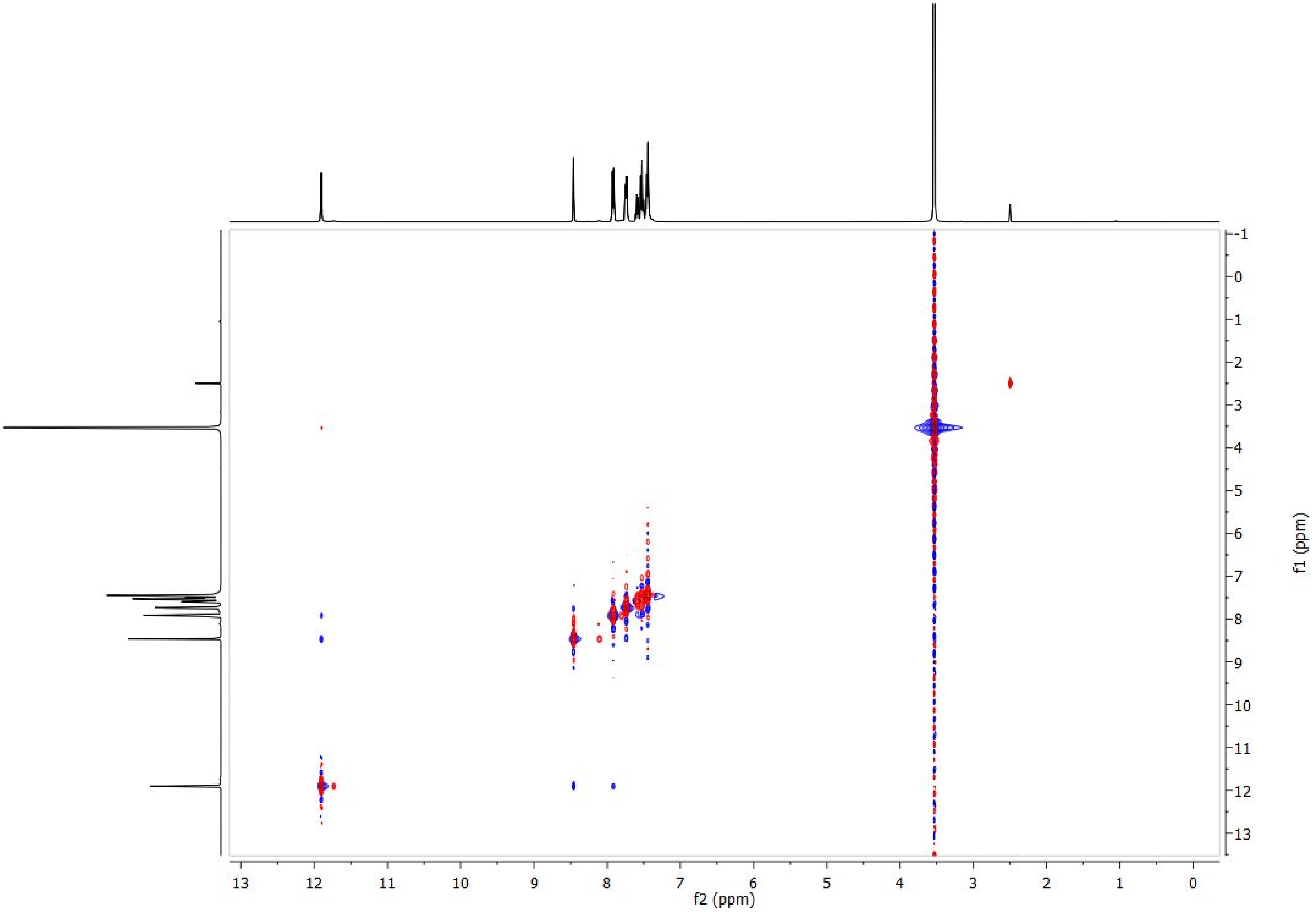

Figure S5 – 2D NOESY NMR spectrum of compound **4**.

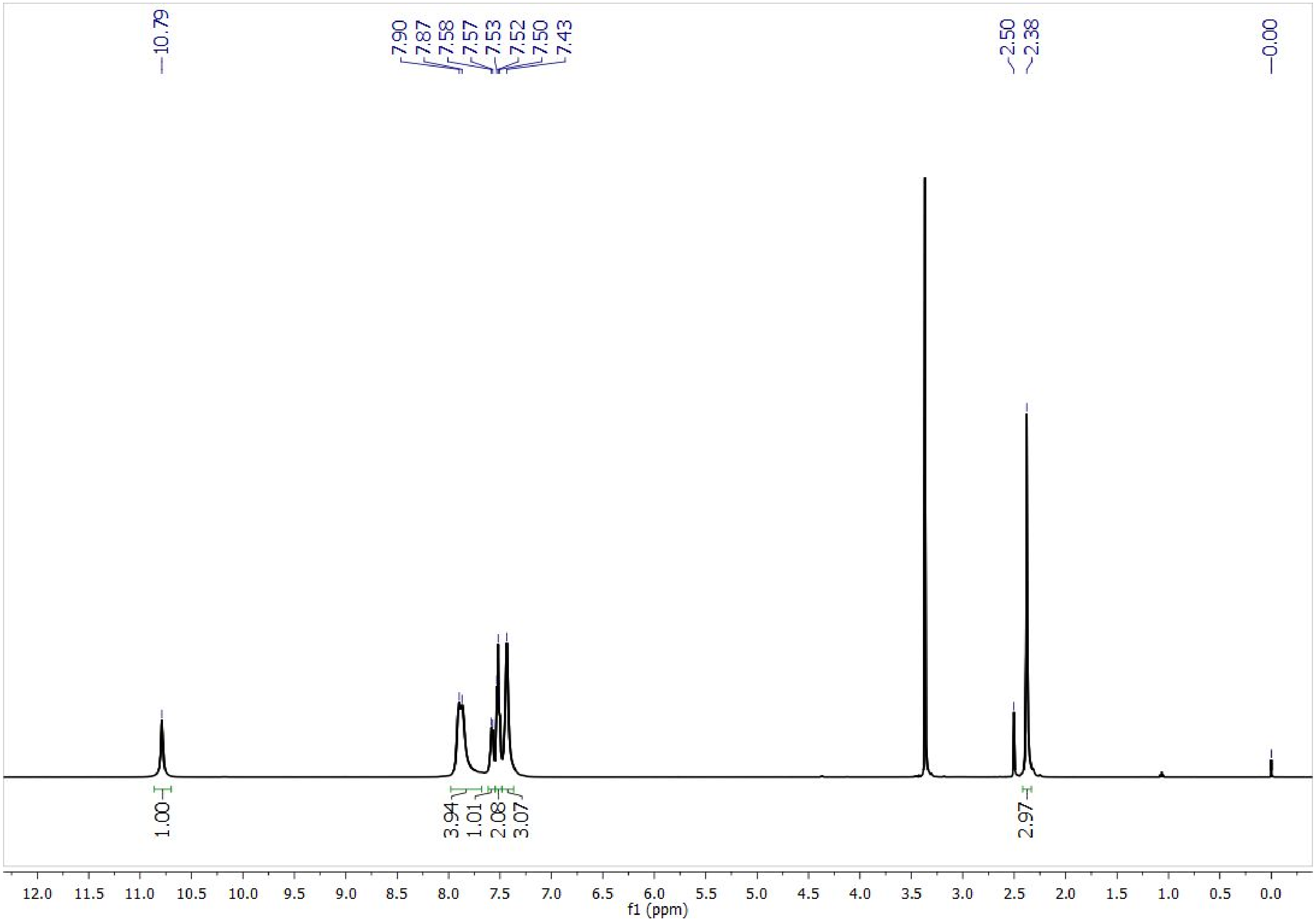

Figure S6 - Compound **5** (^{1}H NMR, 500 MHz, DMSO-d_6, TMS).

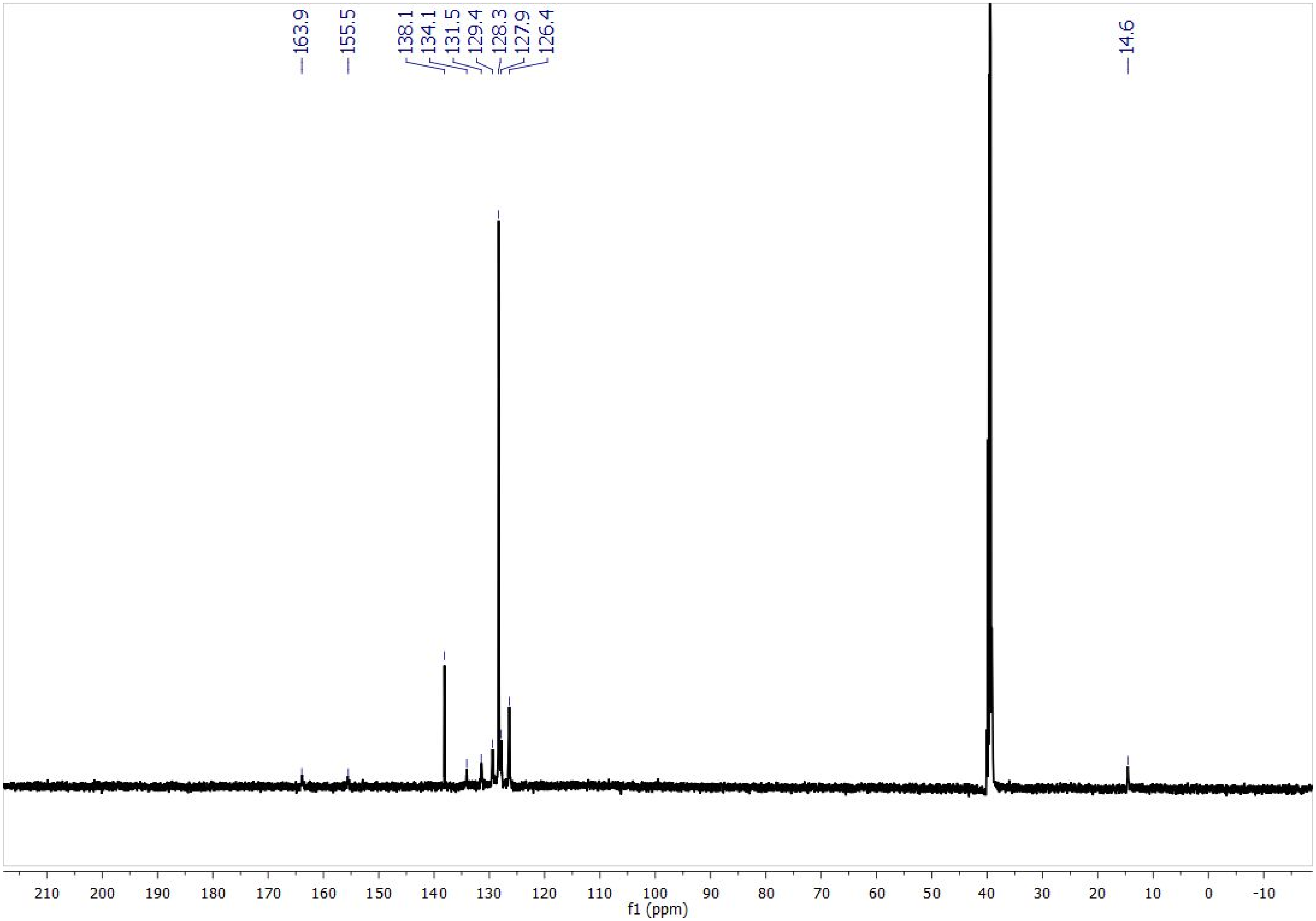

Figure S7 - Compound **5** (^{13}C NMR, 125 MHz, DMSO-d_6, TMS).

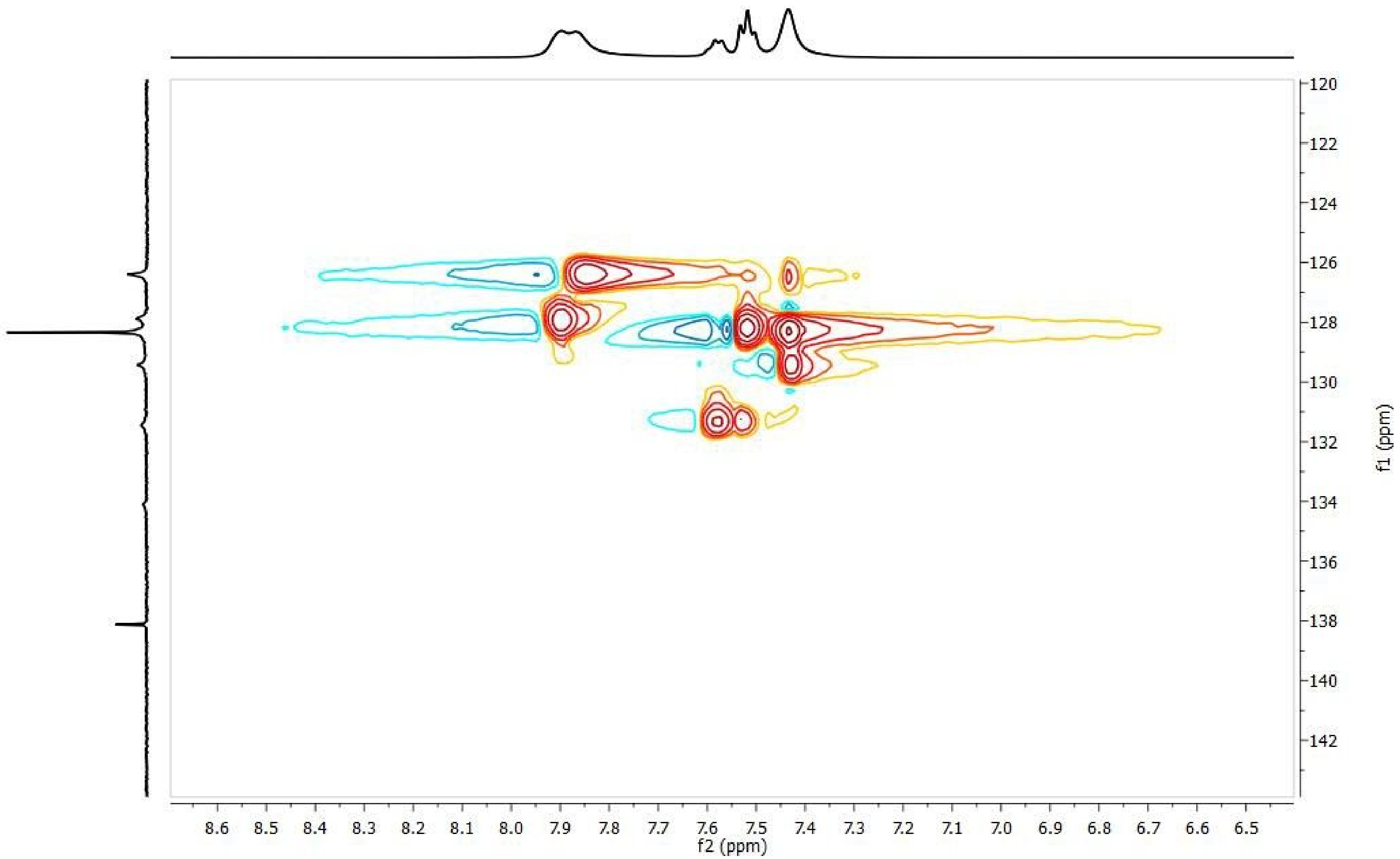

Figure S8 - 2D NMR HSQC spectrum of compound **5.**

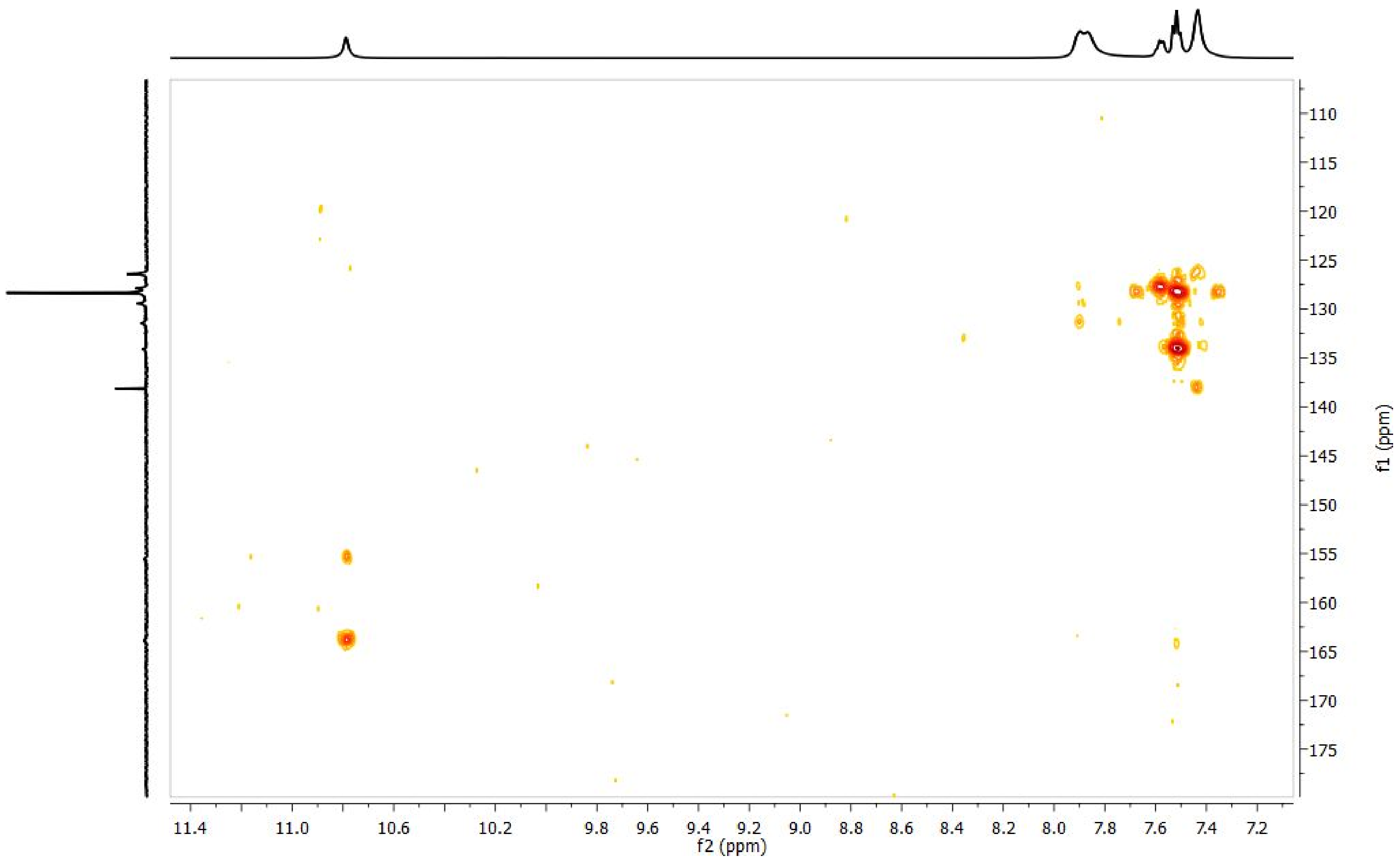

Figure S9 - 2D NMR HMBC spectrum of compound **5.**

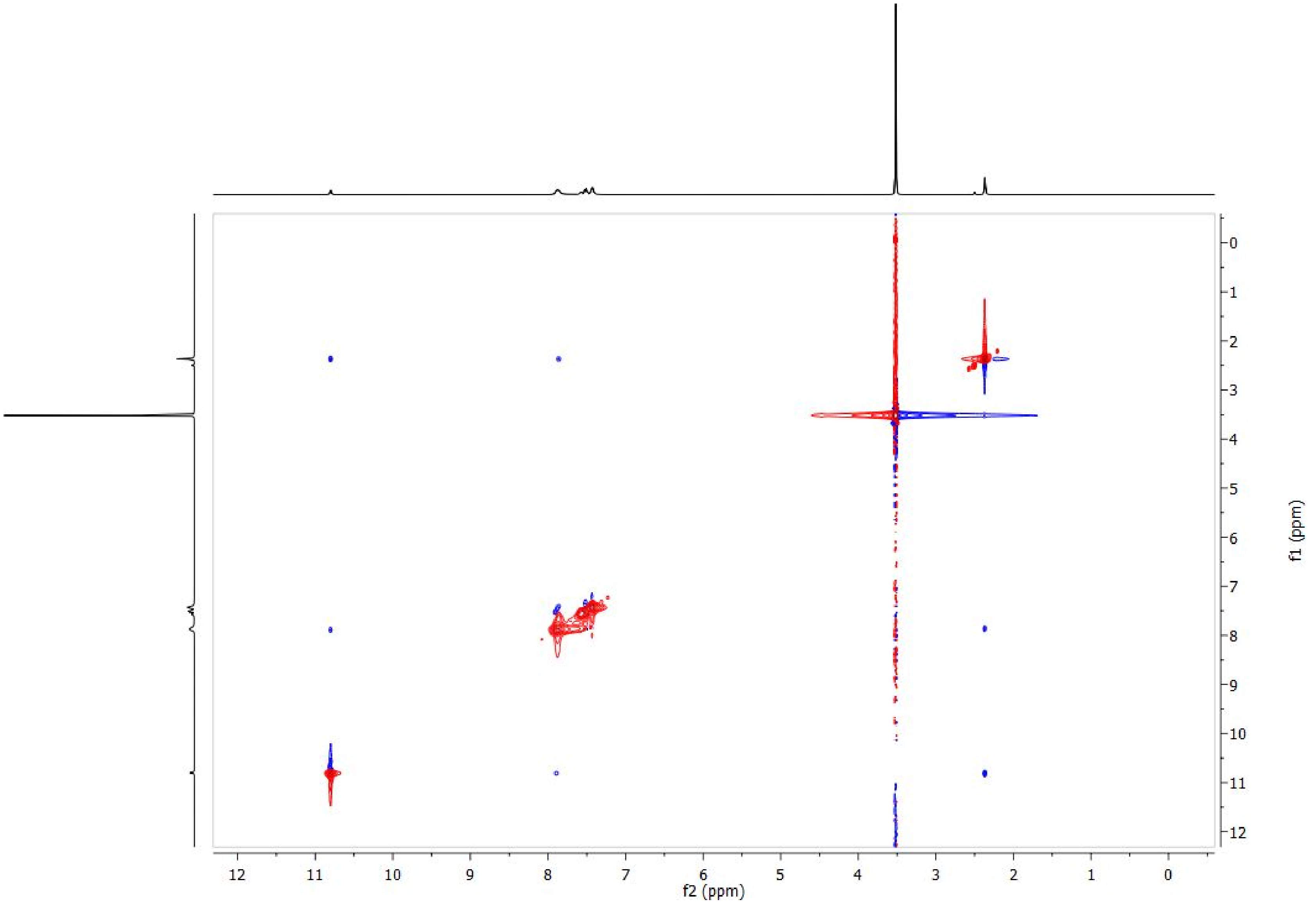

Figure S10 – 2D NOESY NMR spectrum of compound **5**.

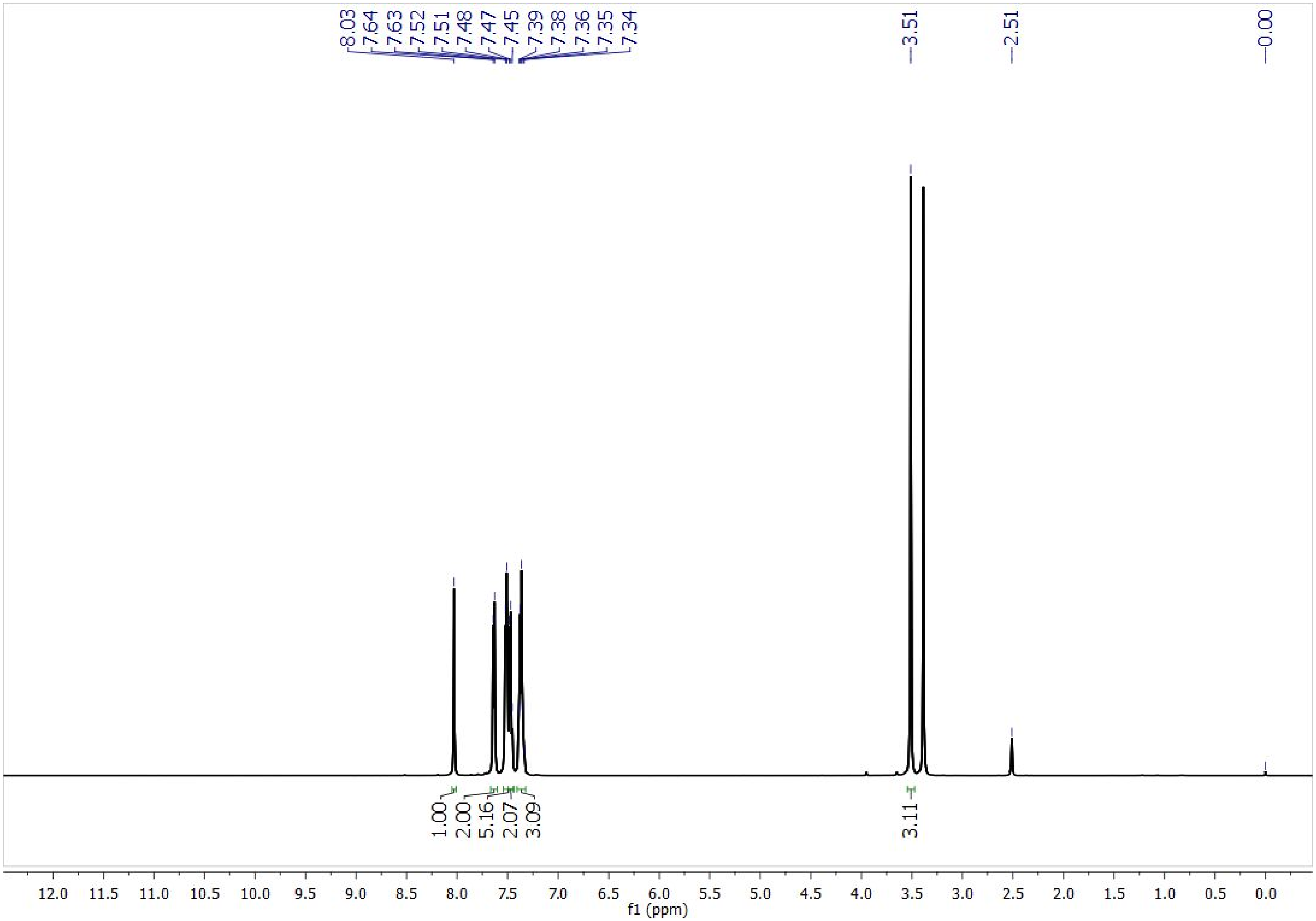

Figure S11 - Compound **6** (^{1}H NMR, 500 MHz, DMSO-d_6, TMS).

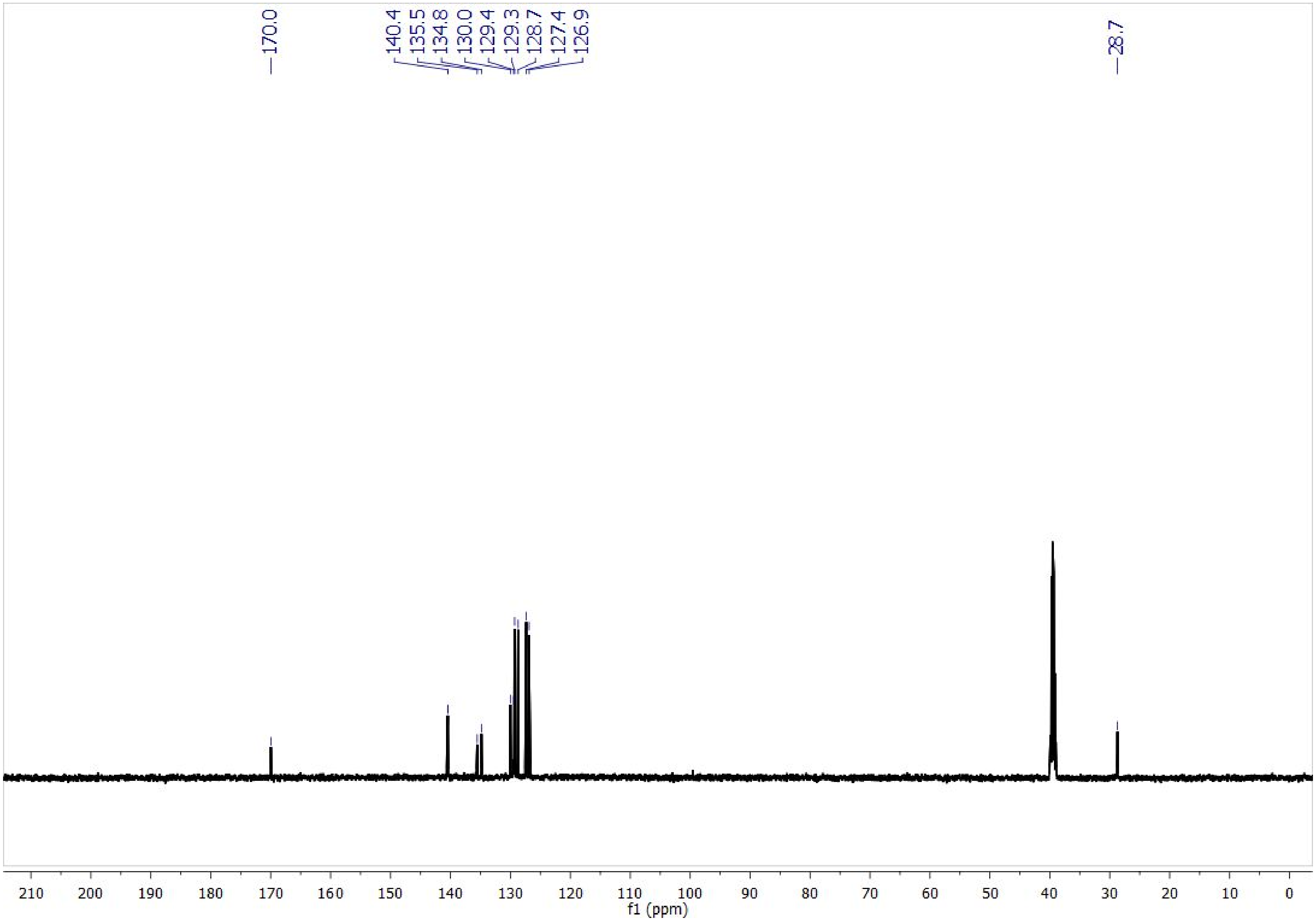

Figure S12 - Compound **6** (^{13}C NMR, 125 MHz, DMSO-d_6, TMS).

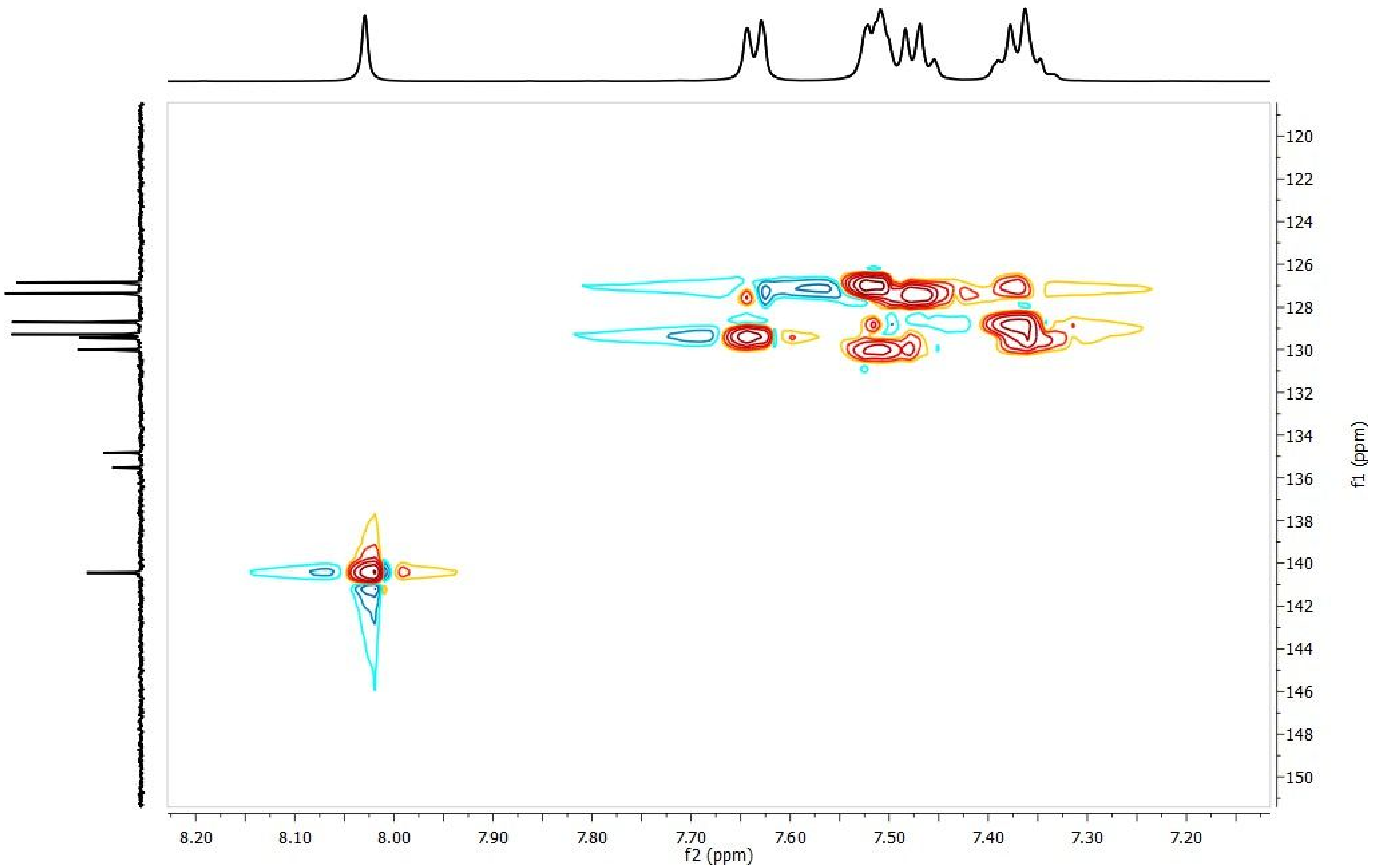

Figure S13 - 2D NMR HSQC spectrum of compound **6.**

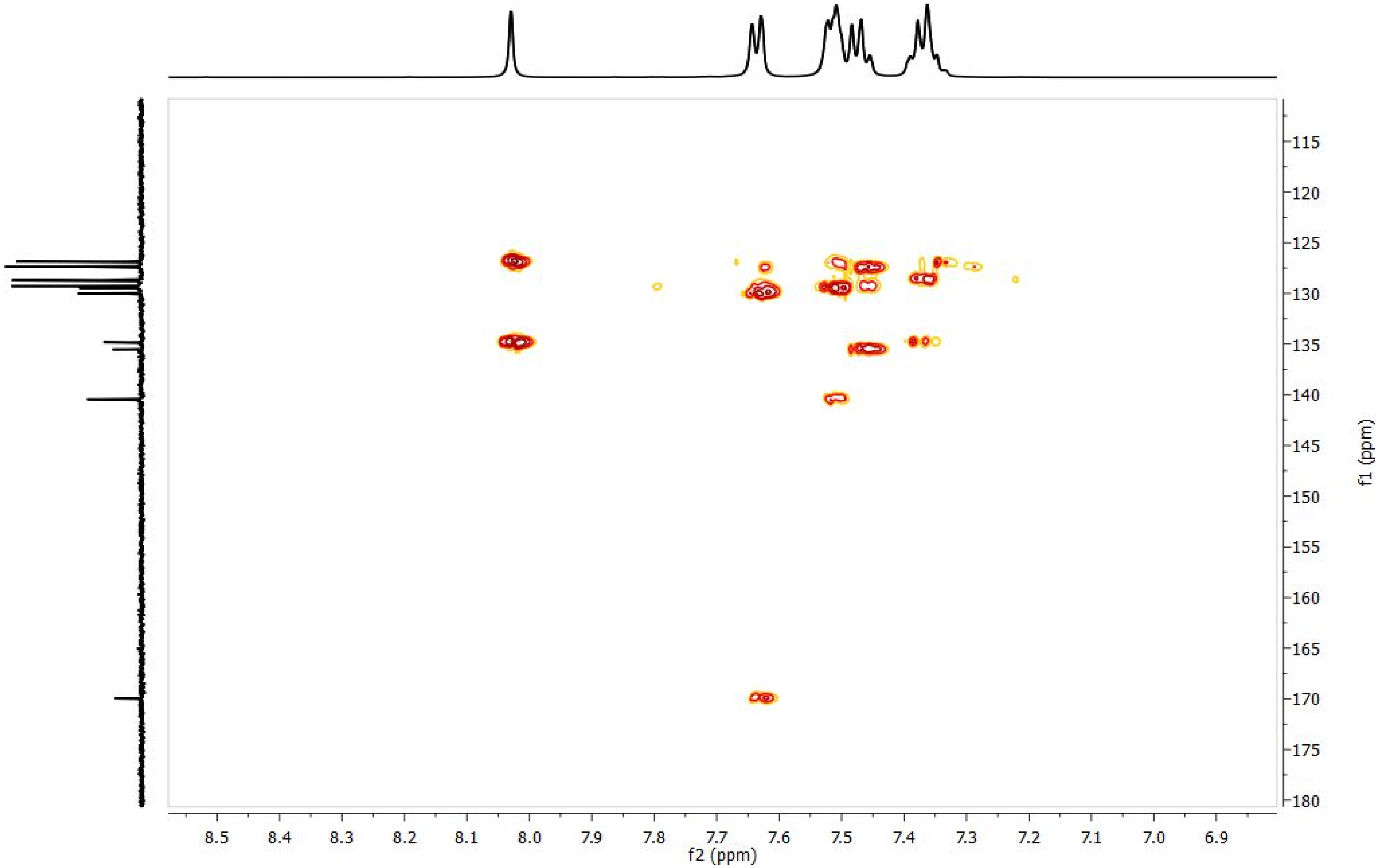

Figure S14 - 2D NMR HMBC spectrum of compound **6.**

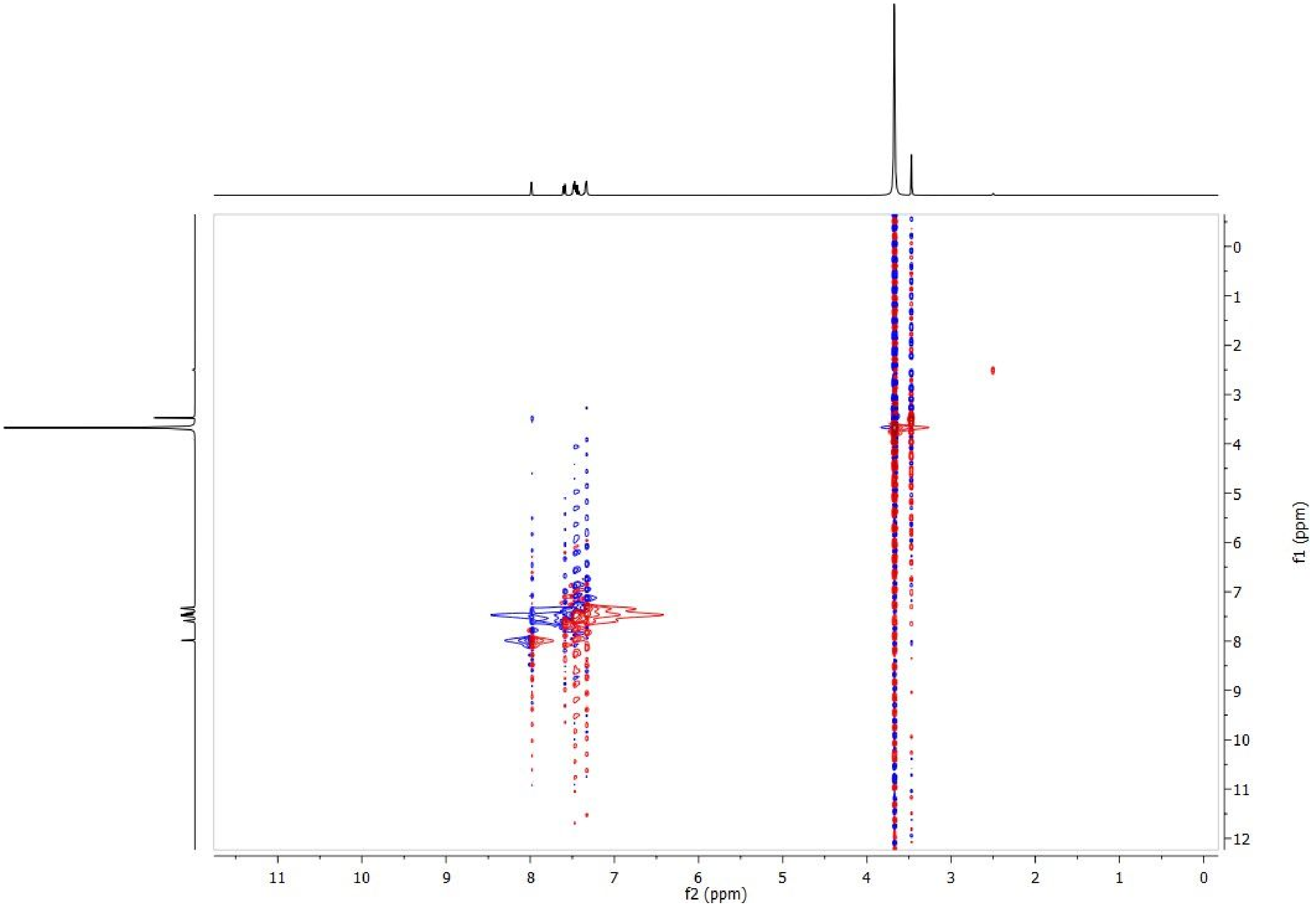

Figure S15 – 2D NOESY NMR spectrum of compound **6**.

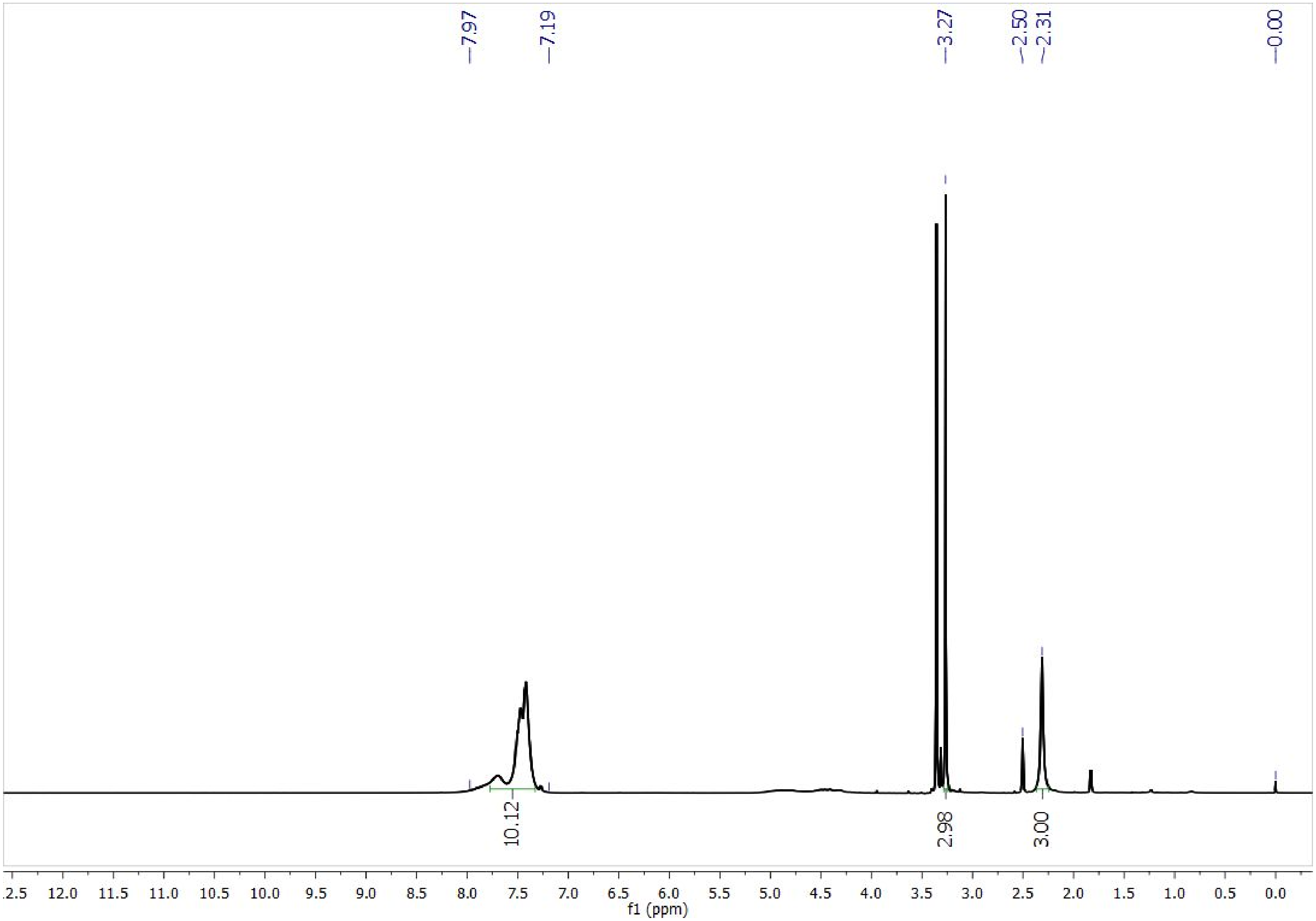

Figure S16 - Compound **7** (^{1}H NMR, 500 MHz, DMSO-d_6, TMS).

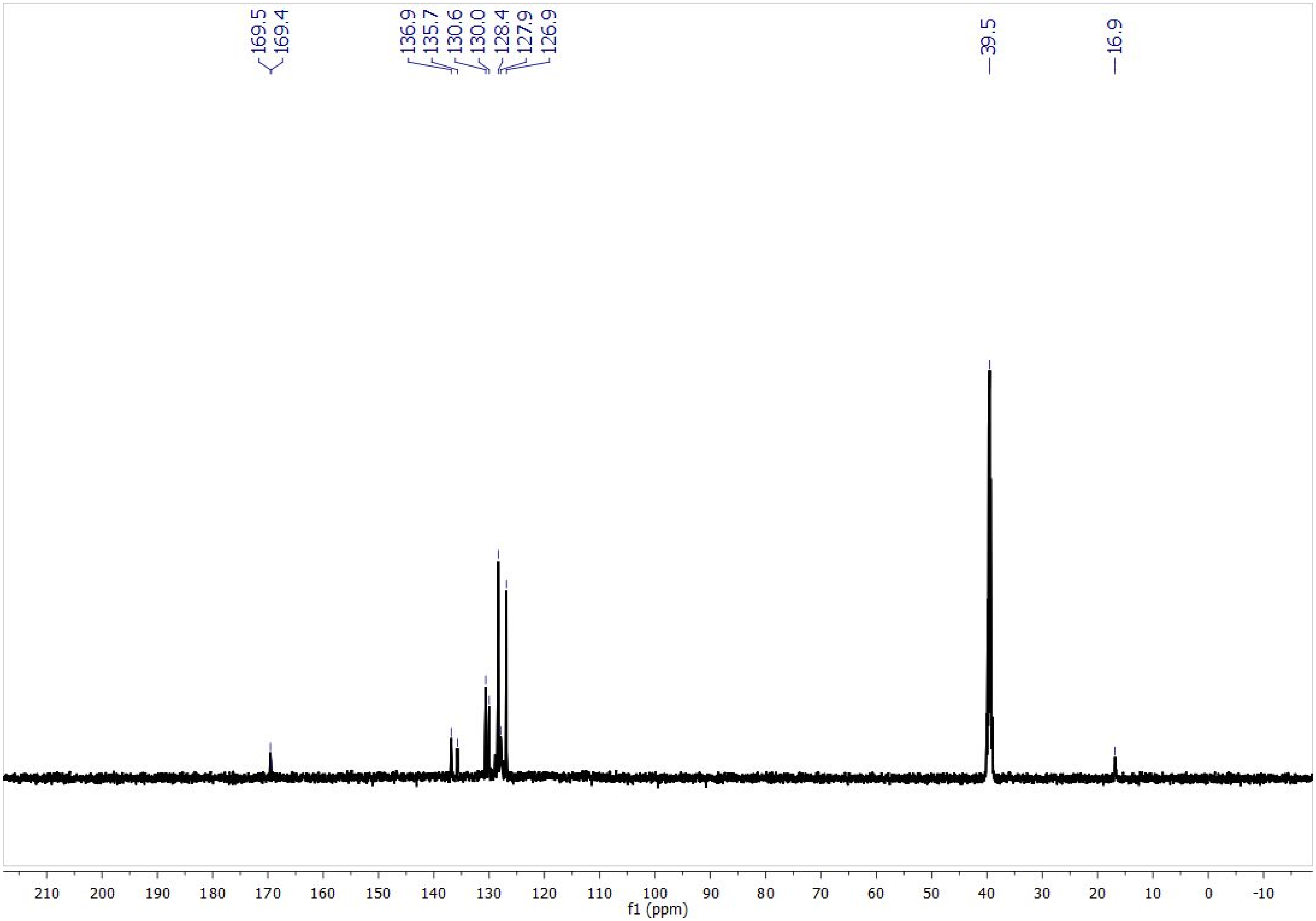

Figure S17 - Compound **7** (^{13}C NMR, 125 MHz, DMSO-d_6, TMS).

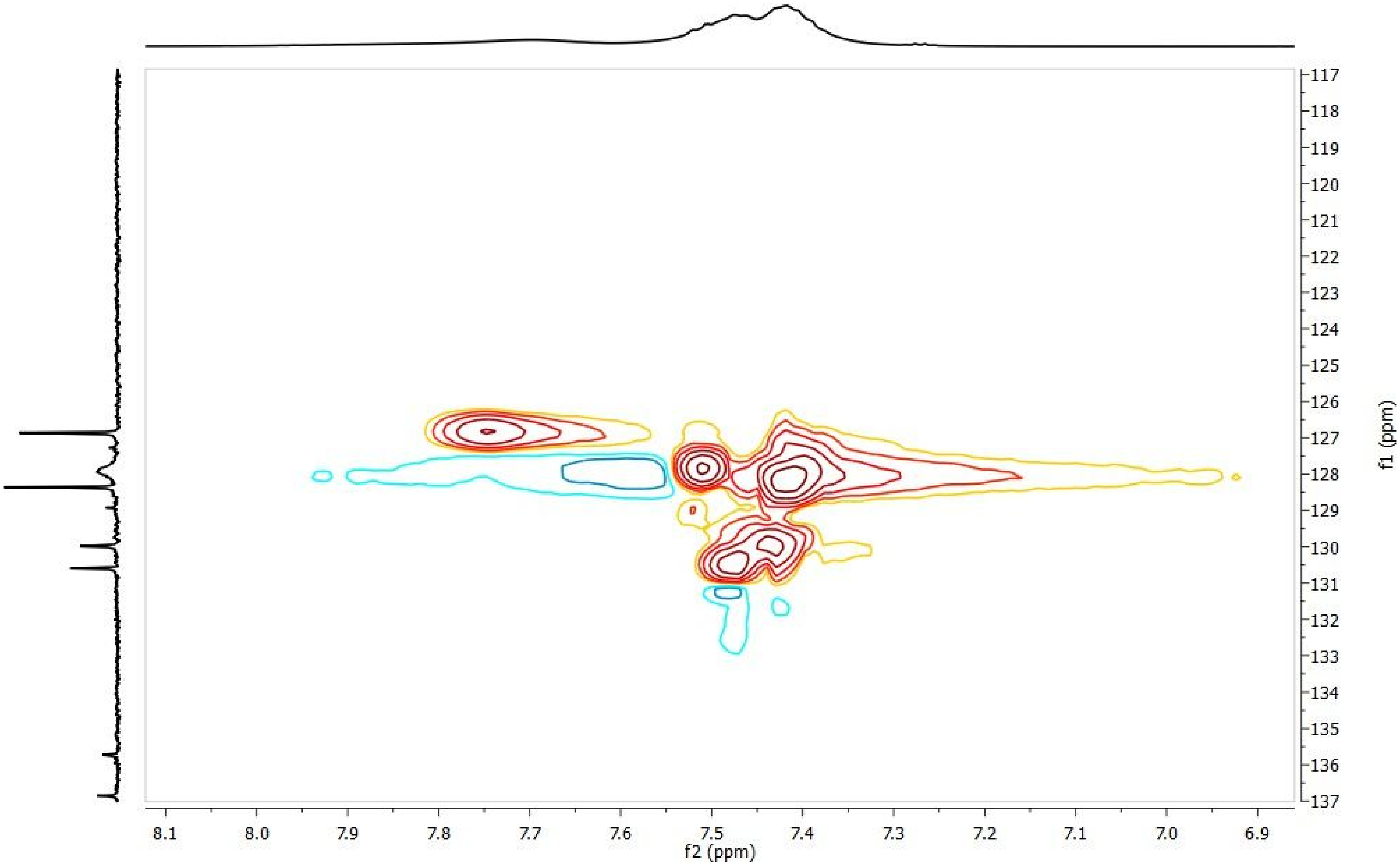

Figure S18 - 2D NMR HSQC spectrum of compound **7.**

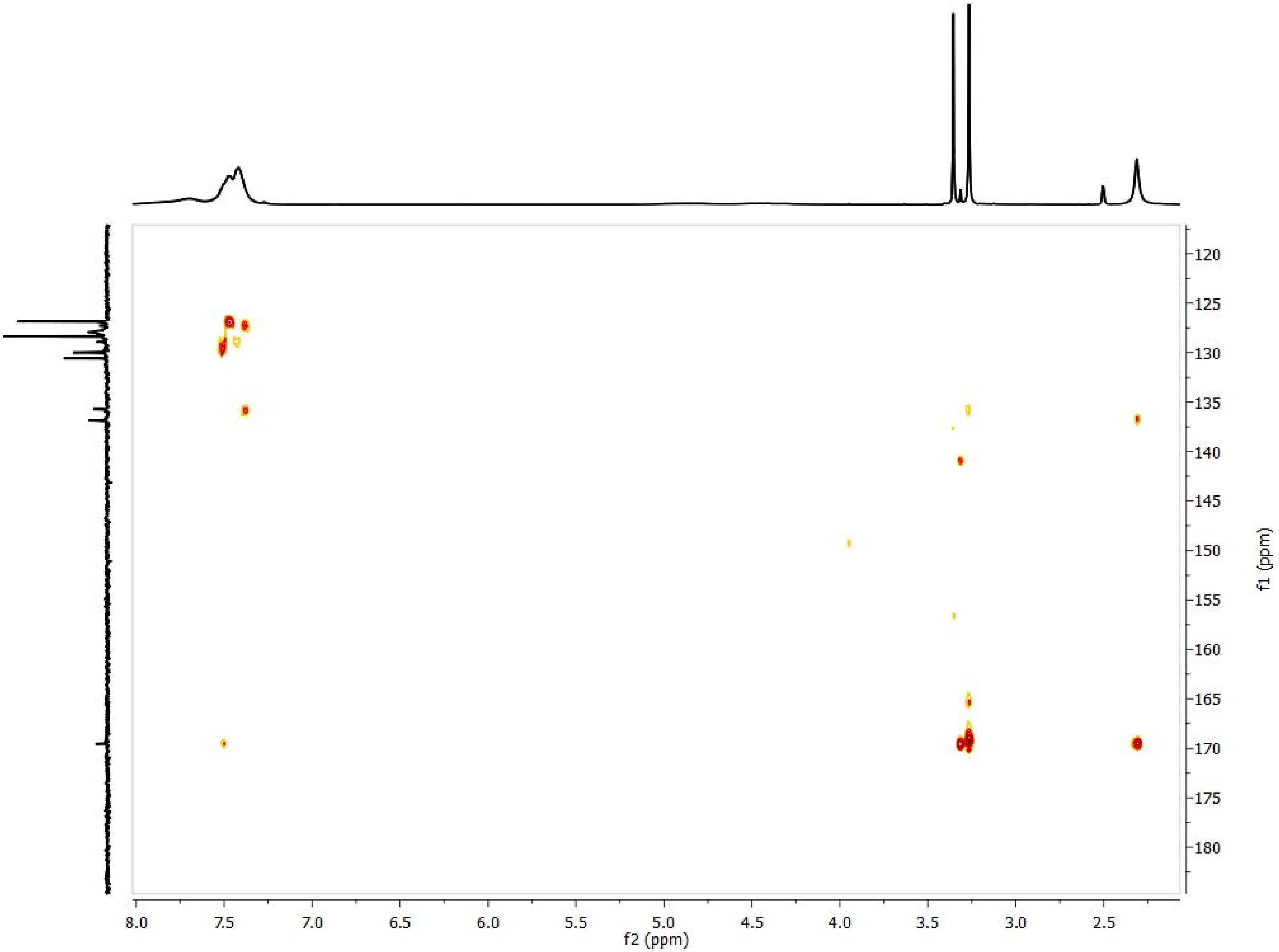

Figure S19 - 2D NMR HMBC spectrum of compound **7.**

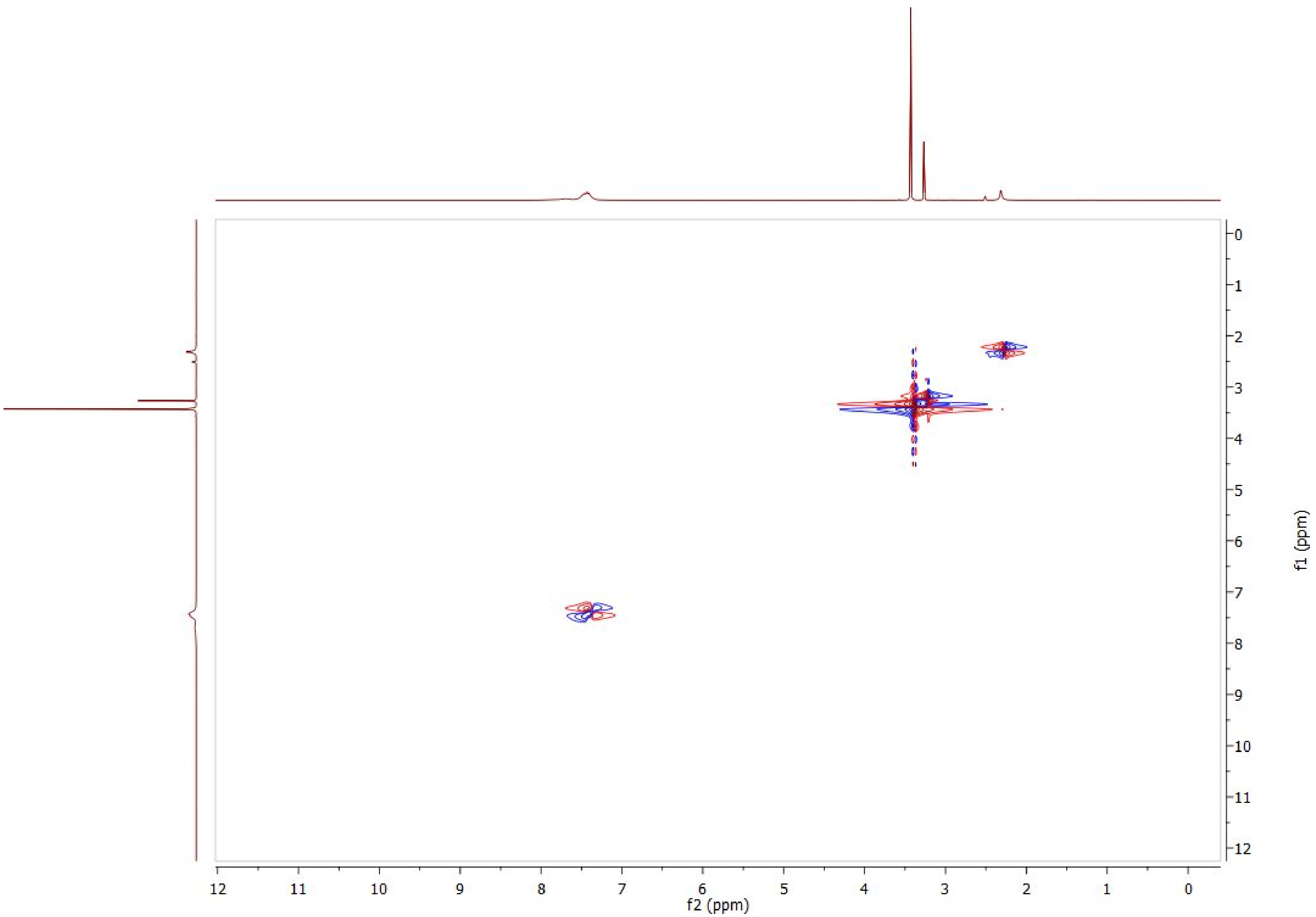

Figure S20 – 2D NOESY NMR spectrum of compound **7**.

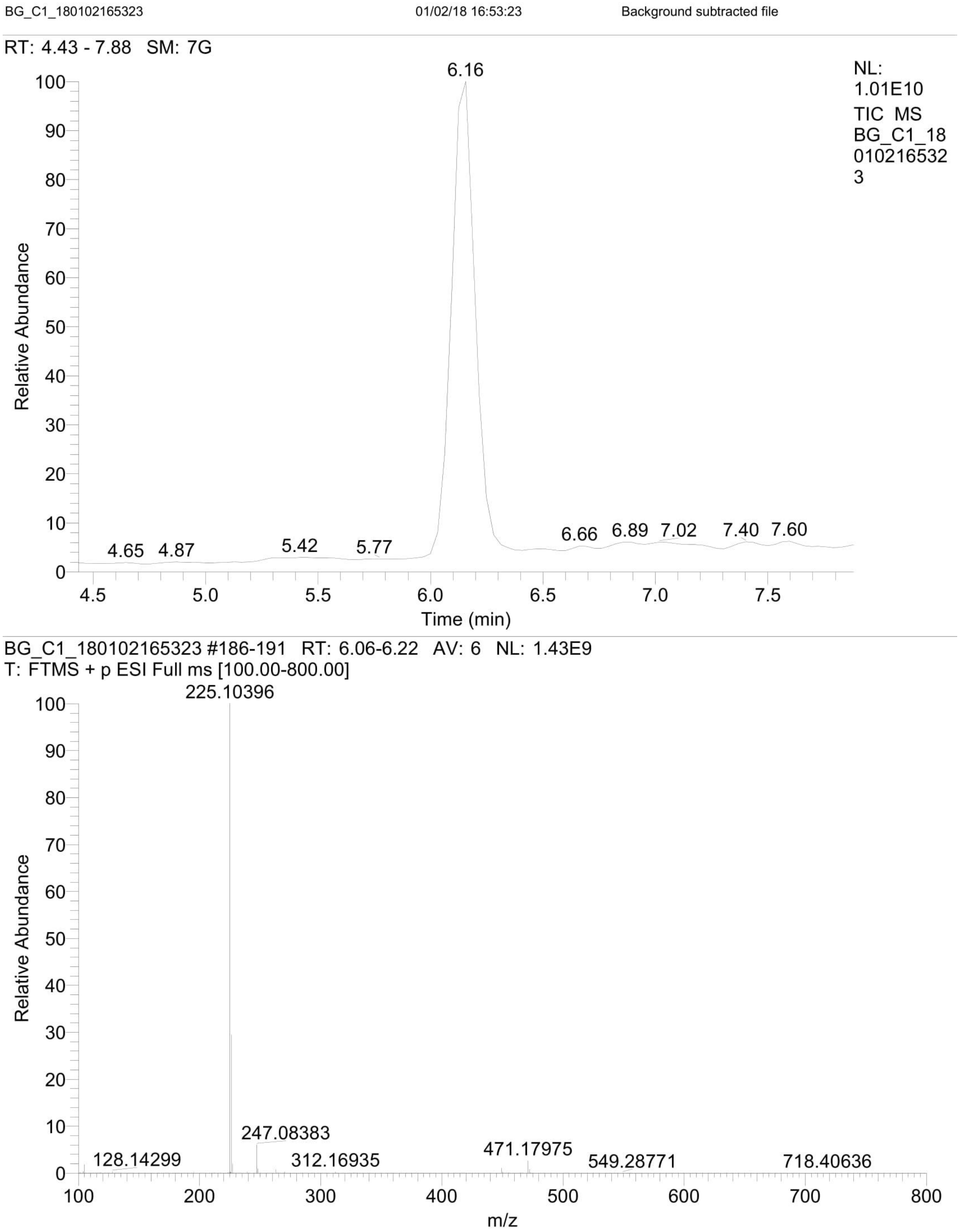

Figure S21 - High-resolution mass spectrometry for compound **4**.

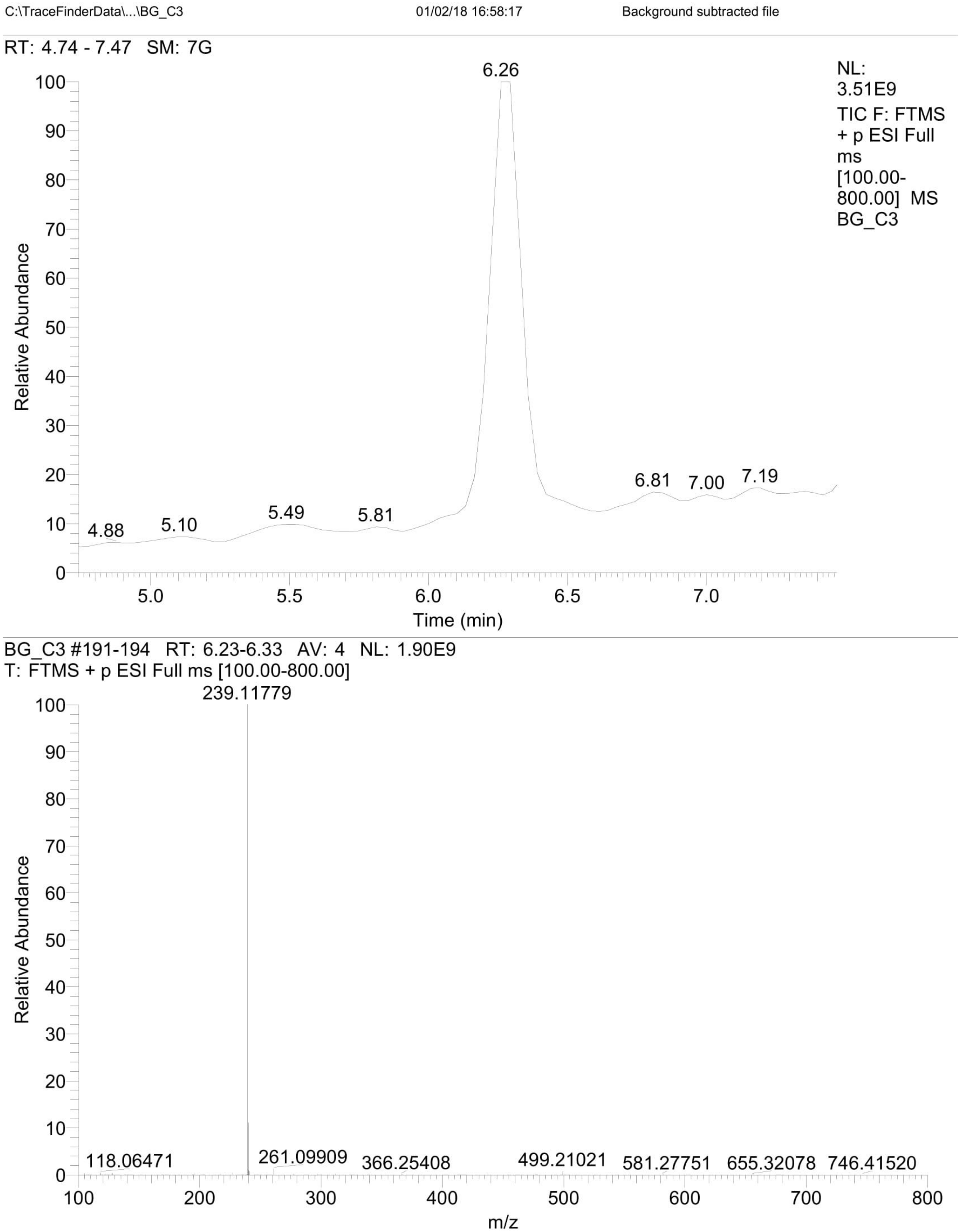

Figure S22 - High-resolution mass spectrometry for compound **5**.

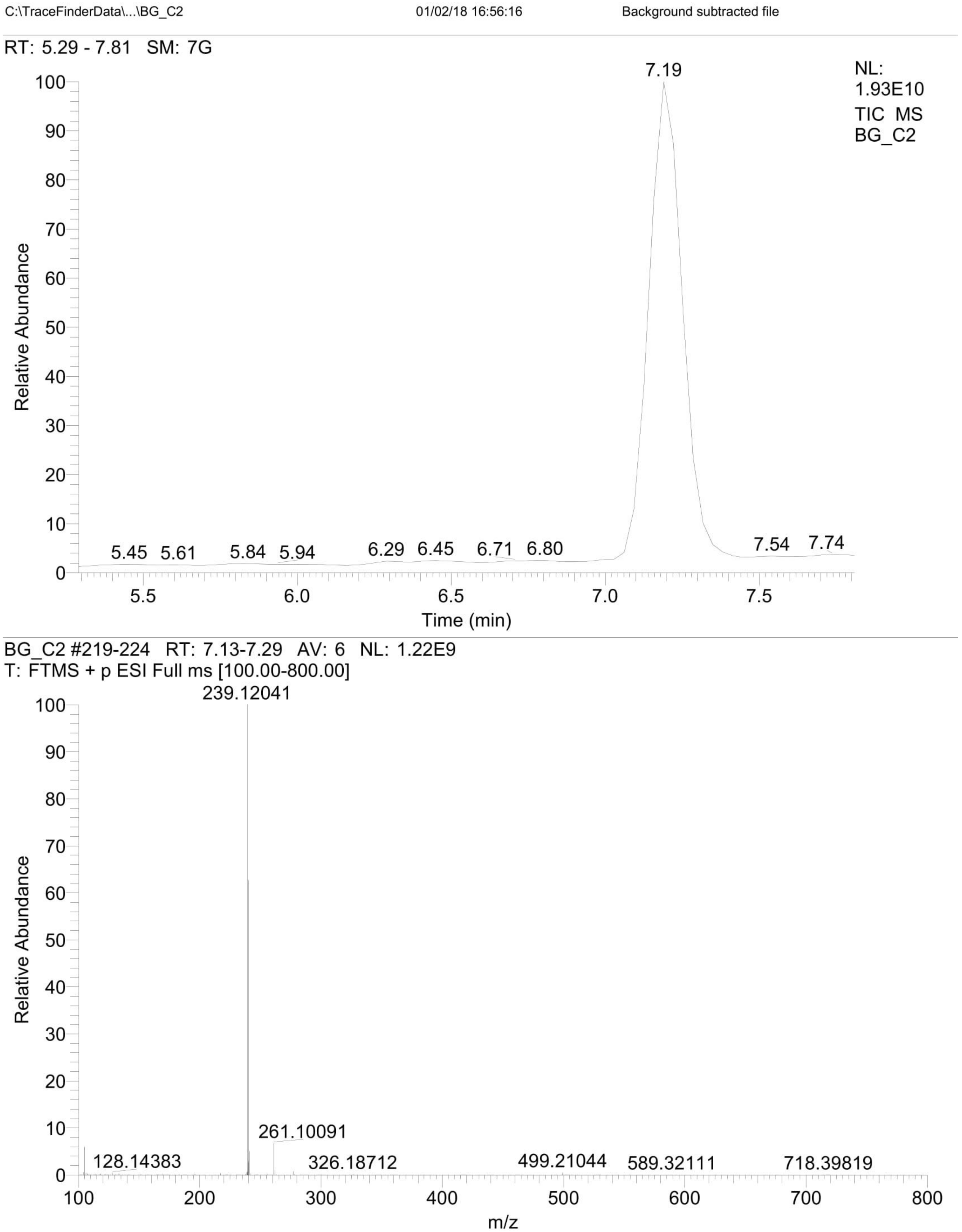

Figure S23 - High-resolution mass spectrometry for compound **6**.

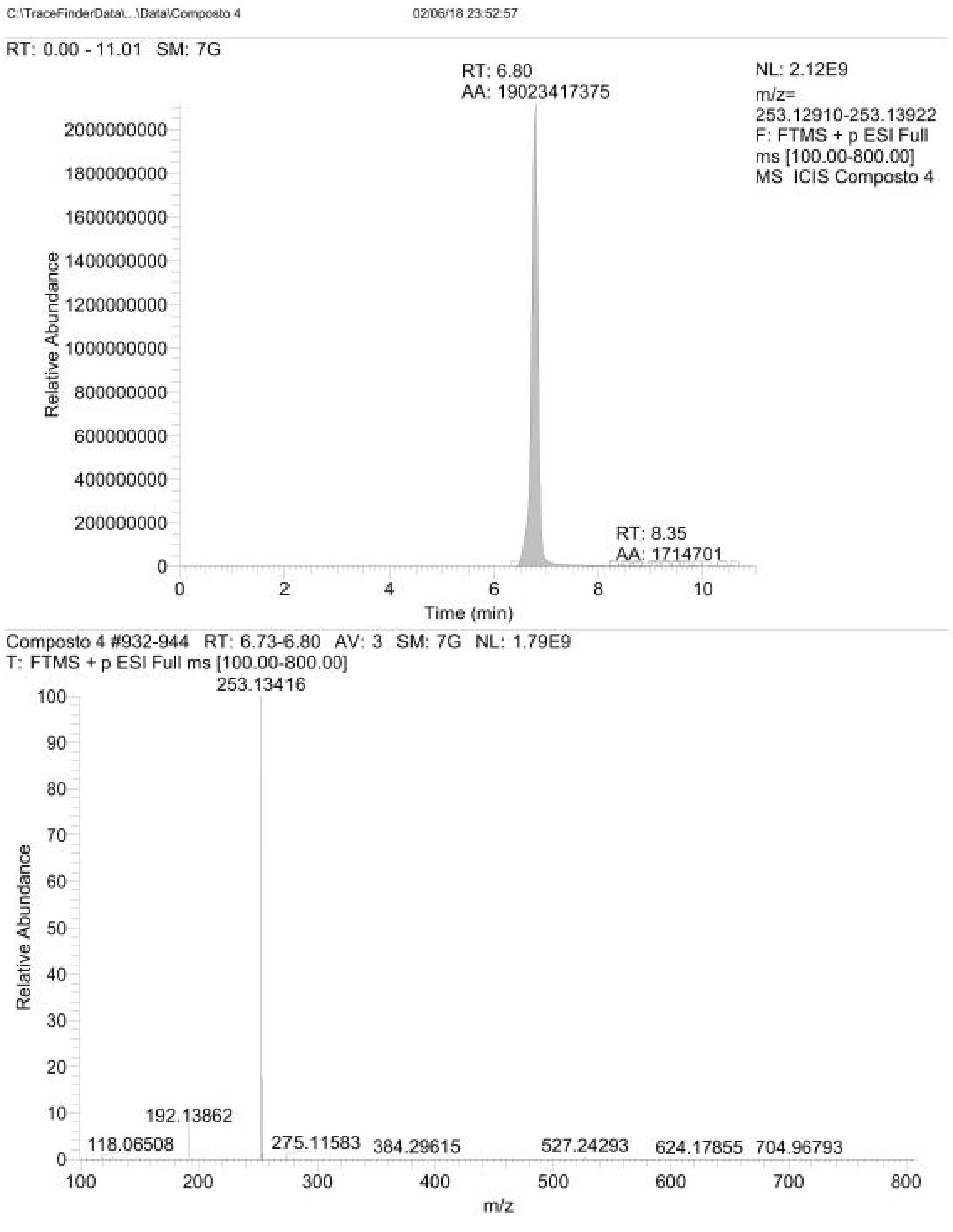

Figure S24 - High-resolution mass spectrometry for compound **7**.

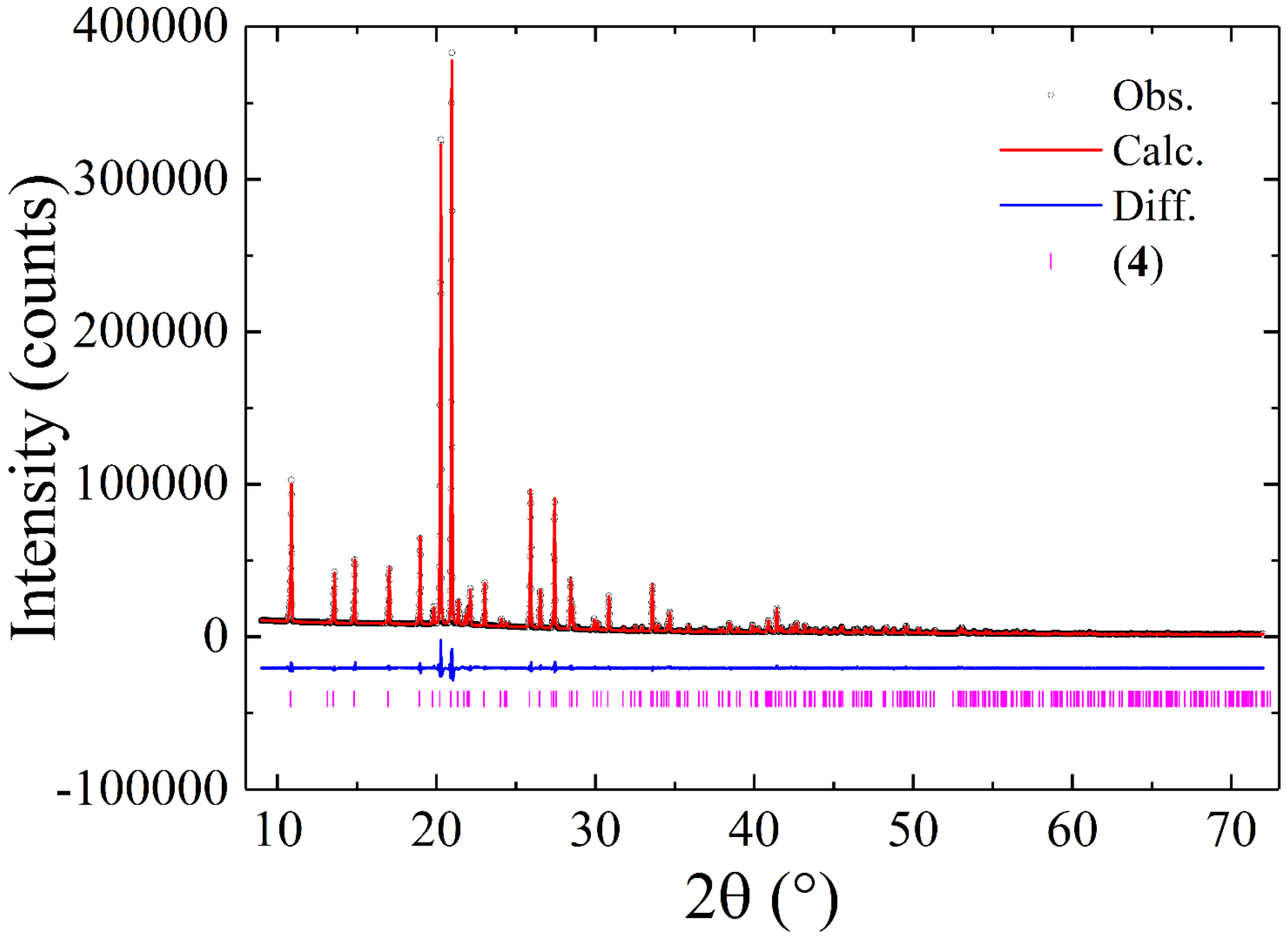

Figure S25 - Plot from the final Rietveld refinement of compound **4**. Black circles represent observed data; the red line indicates the calculated pattern and the blue line at the bottom represents the difference between the observed and calculated patterns. Magenta vertical bars indicate the Bragg reflections.

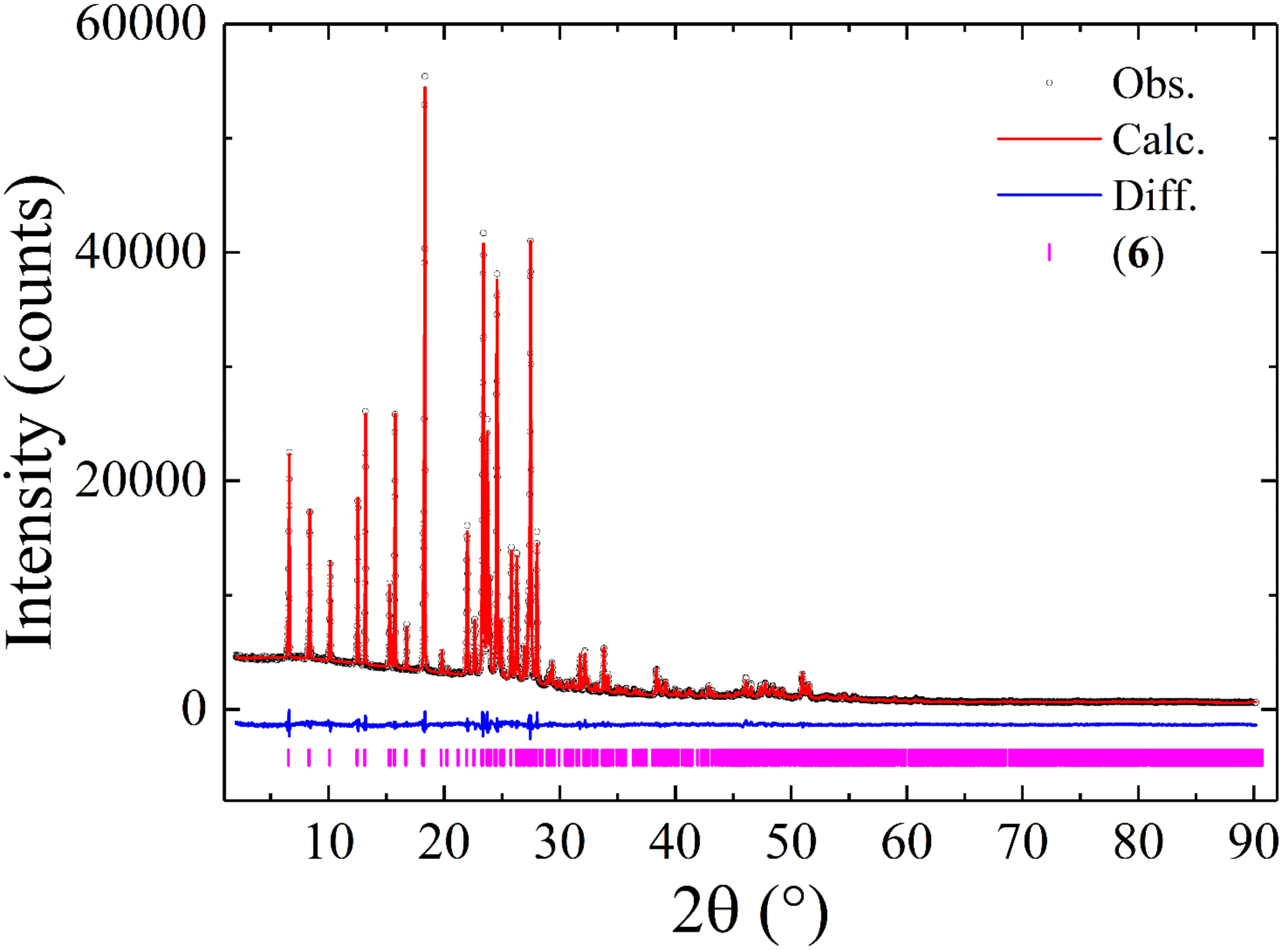

Figure S26 - Plot from the final Rietveld refinement of compound **6**. Black circles represent observed data; the red line indicates the calculated pattern and the blue line at the bottom represents the difference between the observed and calculated patterns. Magenta vertical bars indicate the Bragg reflections.

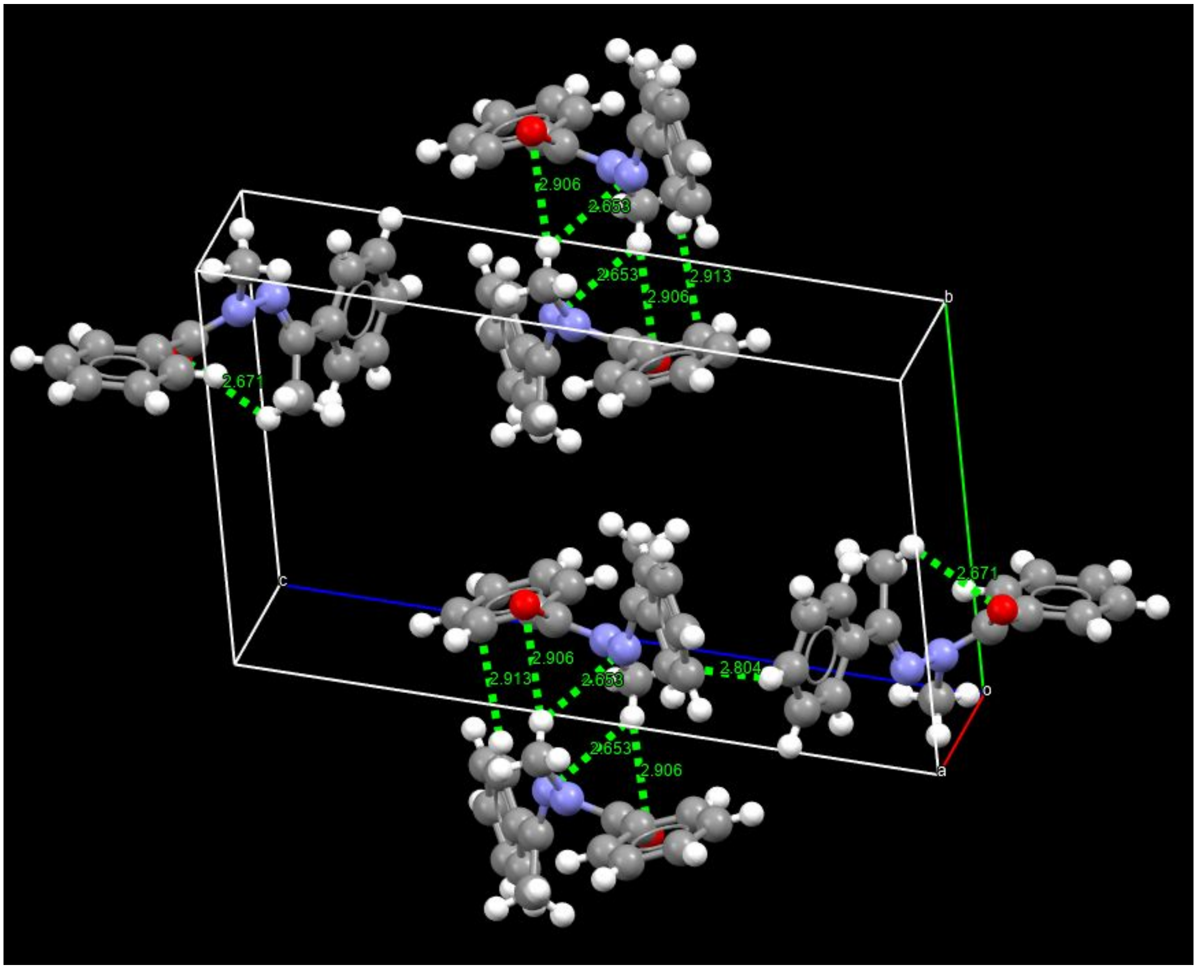

Figure S27 - Crystal packing contacts for compound 7 are highlighted in green, with T-shaped π-π stacking interactions measured at distances of 2.804 Å and 2.913 Å, non-conventional hydrogen bonding between N-C<u>H</u>$_3$ protons and the carbonyl oxygen at distances of 2.671 Å (intramolecular) and 2.906 Å (intermolecular), and with the imine nitrogen at a distance of 2.653 Å.
